# Low physical activity is associated with adverse health outcome and higher costs in Indonesia: A national panel study

**DOI:** 10.3389/fcvm.2022.972461

**Published:** 2022-12-16

**Authors:** Kanya Anindya, Tiara Marthias, Muhammad Zulfikar Biruni, Sophia Hage, Nawi Ng, Anthony A. Laverty, Barbara McPake, Christopher Millett, Tilahun Nigatu Haregu, Emily S. G. Hulse, Yingting Cao, John Tayu Lee

**Affiliations:** ^1^Nossal Institute for Global Health, The University of Melbourne, Melbourne, VIC, Australia; ^2^School of Public Health and Community Medicine, Institute of Medicine, Sahlgrenska Academy, University of Gothenburg, Gothenburg, Sweden; ^3^Directorate of Pharmaceutical Services, Ministry of Health of the Republic of Indonesia, Jakarta, Indonesia; ^4^Royal Sports Performance Center, Jakarta, Indonesia; ^5^Indonesia Sports Medicine Doctor Association, Jakarta Pusat, Indonesia; ^6^Public Health Policy Evaluation Unit, School of Public Health, Imperial College London, London, United Kingdom; ^7^Center for Health Policy, School of Population and Global Health, The University of Melbourne, Melbourne, VIC, Australia; ^8^Non-Communicable Disease Unit, Melbourne School of Population and Global Health, The University of Melbourne, Melbourne, VIC, Australia; ^9^Department of Primary Care and Public Health, School of Public Health, Imperial College London, London, United Kingdom; ^10^College of Health and Medicine, Australian National University, Canberra, ACT, Australia

**Keywords:** physical inactivity, Indonesia, non-communicable diseases, cardiovascular disease, health care utilization, catastrophic health expenditure, work productivity

## Abstract

**Aims:**

To assess the association between low physical activity, cardiovascular disease (CVD) and risk factors, health service utilization, risk of catastrophic health expenditure, and work productivity in Indonesia.

**Methods:**

In this population-based, panel data analysis, we used data from two waves of the Indonesian Family Life Survey (IFLS) for 2007/2008 and 2014/2015. Respondents aged 40–80 years who participated in both waves were included in this study (*n* = 5,936). Physical activity was assessed using the International Physical Activity Questionnaire (IPAQ-SF). Multinomial logistic regression model was used to examine factors associated with physical activity levels (low, moderate, and high). We applied a series of multilevel mixed-effect panel regression to examine the associations between physical activity and outcome variables.

**Results:**

The prevalence of low physical activity increased from 18.2% in 2007 to 39.6% in 2014. Compared with those with high physical activity, respondents with low physical activity were more likely to have a 10-year high CVD risk (AOR: 2.11, 95% CI: 1.51–2.95), use outpatient care (AOR: 1.26, 95% CI: 1.07–1.96) and inpatient care (AOR 1.45, 95% CI: 1.07–1.96), experience catastrophic health expenditure of 10% of total household expenditure (AOR: 1.66, 95% CI: 1.21–2.28), and have lower labor participation (AOR: 0.24, 95% 0.20–0.28).

**Conclusions:**

Low physical activity is associated with adverse health outcomes and considerable costs to the health system and wider society. Accelerated implementation of public health policies to reduce physical inactivity is likely to result in substantial population health and economic benefits.

## Introduction

Physical inactivity is a major risk factor for non-communicable diseases (NCDs), which are responsible for 78% of mortality in low-and middle-income countries (LMICs) (1). Indonesia, the third most populous LMIC (after China and India), is undergoing rapid economic and demographic transitions (2–4), leading to an increase in sedentary lifestyles and physical inactivity. A recent nationwide survey revealed that the prevalence of low physical activity in the population aged 10 years and over increased from 26.1% in 2013 to 33.5% in 2018 (5, 6). Indonesia has also experienced a substantial increase in the prevalence of NCDs between 1990 and 2015, where the total disability-adjusted life year (DALYs) increased by 10.5, 30.2, and 54.9% for ischemic heart disease, cerebrovascular disease and diabetes, respectively (7).

High level evidence reviews have found that physical inactivity is associated with adverse health outcomes, higher health care utilization and productivity loss (8, 9). However, studies looking at the economic burden of physical inactivity in LMICs is sparse. A recent systematic review by Ranasinghe et al. examined the economic burden of physical activity in LMICs and found that 12 out of 18 studies were conducted in Brazil and China, with no studies from Indonesia (9). To fill this gap, using longitudinal data, this study examines: (1) the prevalence of low physical activity across sociodemographic groups between 2007 and 2014; (2) factors associated with low physical activity; (3) the associations between physical activity and cardiovascular diseases (CVDs) and risk factors; (4) the associations between physical activity, health service utilization, and catastrophic health expenditure; (5) the relationships between physical activity and work productivity.

## Methods

This study utilizes two waves of longitudinal data from the Indonesian Family and Life Survey (IFLS) 2007/2008 (IFLS-4) and 2014/2015 (IFLS-5). IFLS is an ongoing longitudinal survey initiated in 1993 that includes modules on physical activity, health care utilization and expenditure. The original sampling frame was based on 13 out of 27 provinces in 1993, representing 83% of the population. Data collection of the IFLS-5 was conducted between September 2014–March 2015, with 76% re-contact rate from the IFLS-1. A detailed description of the survey methods published elsewhere (10, 11). Following WHO CVD risk chart's study participants, we included individuals aged 40–80 years who participated in both waves. After excluding respondents with missing values in outcome variables and covariates (4.3% in IFLS-4 and 9.4% in IFLS-5), our final sample consists of 5,936 respondents (Supplementary Figure A1).

### Patient and public involvement

As this study used secondary dataset that are publicly available, there was no direct patient involvement in the design and implementation of the study.

### Variables

#### Physical activity

Our main independent variable was the level of physical activity, which represents the respondent's amount of time spent on different types of physical activity. Physical activity was assessed using the modified version of the short form of the International Physical Activity Questionnaire (IPAQ-SF) (12). The IPAQ is one of the most commonly used instruments and is recognized internationally as a reliable population-based measurement for physical activity. The IPAQ collects types and duration of physical activity that individuals engaged in for the last 7 days. Based on intensity level, activities were divided into three domains, including walking, moderate-intensity (e.g., carrying light loads, bicycling at a regular pace, or mopping the floor), and vigorous-intensity (e.g., heavy lifting, digging, aerobics, fast bicycling, cycling with loads). The total duration of activities, recorded in minutes and days, was transformed to Metabolic Equivalent of Tasks (METs)-minutes. We categorized respondents into high, moderate and low physical activity following the IPAQ scoring guidelines as shown in Table 1 (12). Further detail of the calculation is available in Supplementary Table A1.

**Table 1:** Categorization of physical activity levels.

**Category**	**Criteria**
High	a) Vigorous-intensity activity on at least 3 days achieving ≥1,500 MET-minutes/week, or
	b) 7 or more days of any combination of walking, moderate-intensity or vigorous-intensity activities achieving ≥3,000 MET-minutes/week.
Moderate	a) 3 or more days of vigorous-intensity activity of at least 20 min per day, or
	b) 5 or more days of moderate-intensity activity and/or walking of at least 30 min per day, or
	c) 5 or more days of any combination of walking, moderate-intensity or vigorous intensity activities achieving ≥600 MET-minutes/week.
Low	Individuals who *did* not meet criteria for moderate and high physical activity are considered “low”.

Classification of physical activity level based on International Physical Activity Questionnaire (IPAQ).

#### Health outcomes

The health outcomes assessed include: (i) CVDs status and the risk factors (diabetes, obesity, hypertension); (ii) the 10-year CVD risk score using the 2019 WHO CVD risk charts. Respondents were asked whether they had been diagnosed with any of the following CVDs (coronary heart disease, stroke, or other atherosclerotic diseases) or the risk factors (hypertension, overweight/obesity, and diabetes). Physical examination was performed to measure hypertension (systolic blood pressure of ≥140 mmHg and/or diastolic blood pressure of ≥90 mmHg) and overweight/obesity status (body mass index/BMI ≥ 23 jg/m2) (13, 14). Furthermore, those who were taking hypertension or diabetes medication at the time of survey were classified as having hypertension or diabetes, respectively. Second, we also constructed 10-year CVD risk scores based on the 2019 WHO CVD risk charts (Southeast Asia) to predict the 10-year risk of a fatal or non-fatal major CVDs event in Indonesia (15). As information on blood cholesterol was not collected in Wave 5, we used a non-laboratory-based prediction model which considers age, tobacco use, gender, systolic blood pressure, and BMI to derive the risk categories. The 10-year CVD risk score in this study are categorized as low risk (5 to <10%), moderate risk (10 to <20%), and high risk (≥20%).

#### Health service use and financial burden outcome

Respondents were asked whether or not they had any outpatient visits in the past 4 weeks or inpatient visit last year, the frequency of the visit(s), and the out-of-pocket expenditure (OOPE) incurred during the visits. To estimate the catastrophic effects of OOPE, we used three different thresholds which have been proposed by SDG 3.8.2. and WHO: (1) 10% of total household expenditure; (2) 25% of total household expenditure; and (3) 40% of non-food consumption. OOPE was considered as catastrophic health expenditure (CHE) when OOPE in the last month was equal or exceeding the chosen threshold (16).

#### Work productivity outcomes

The work productivity was measured by assessing labor participation, the number of days of primary activity missed due to poor health, and number of days confined to a bed. Labor participation was determined by the employment status of the respondent at the time of interview, while the latter two variables of productivity loss were derived from self-reported health conditions of poor health within four weeks. The details of the variables are presented in Supplementary Table A1.

#### Covariates

Socioeconomic and demographic variables included were: sex, age group, marital status, ethnicity (Javanese, Sundanese, and others), education level, type of residency (rural, urban), region of residence (Java & Bali, Sumatra, Nusa Tenggara, Kalimantan, and Sulawesi), per capita household expenditure quantiles (Q1 to Q5), health insurance coverage, tobacco-use, food consumption variables. In measuring the association between physical activity and health service utilization, financial burden, and work productivity, we also included two additional covariates: BMI and number of NCDs (single NCD, two NCDs, three or more NCDs). The details of the variables are presented in Supplementary Table A1.

### Statistical analysis

Multinomial logistic regression model was used to examine factors associated with physical activity levels (low, moderate, and high). We applied a series of multilevel mixed-effect regression models to examine the associations between physical activity and outcome variables. A multilevel model for panel data was chosen to consider the nature of the IFLS dataset, i.e., observations are nested within individuals (level one), and individuals were nested within households (level two) (17). This approach was utilized so that the clustering effect of household and individual can be taken into account to obtain better estimates across population characteristics. The multilevel model was derived using unweighted data since this study aims to test the association between physical activity and outcomes within mixed-effects, rather than producing nationally representative estimates (18). A multilevel logistic regression is used to assess the association between physical activity and health outcomes and CHE. The association, adjusted by the covariates, was presented as adjusted odds ratio (AOR) and 95% confidence interval. We also measured intra-class coefficients (ICC) to quantify the total variance explained by individual-level and household-level. Meanwhile, the numerical outcomes (e.g., frequency of health service utilization) were estimated using multilevel negative binomial models due to the overdispersion of the variables (likelihood-ratio test of α = 0, *p* < 0.001) (19). We present the results of multilevel negative binomial models using incidence rate ratio (IRR), interpreted as the estimated rate ratio (e.g., outpatient visit) for one unit change in the independent variable (moderate or low physical activity) relative to a reference category (high physical activity), given the other variables are held constant in the model.

To assess the potential for reverse causation, a robustness check was conducted by rerun our regression model but excluding participants who had limitation in activities of daily living (ADLs) in IFLS-4 or IFLS-5 (20, 21). We defined participants with limitation if they answered “unable to do it” in at least one (1+ADL) of the 15 physical functioning variables (22). The details of generating 1+ADL are presented in Supplementary Table A1. All monetary values were adjusted for purchasing power parity (PPP) and converted to 2014 US Dollars (23). All data were analyzed by using Stata version 14.2 SE.

## Results

### Sample characteristic and prevalence of physical activity levels

The median age in 2007 was 50 years (IQR 45–57), 54.7% were female, 83.7% were married, 14.5% had at least secondary education level or above, 37.5% lived urban area, and 26.9% had health insurance coverage. In 2014, the median age was 57 years (IQR 51–64), 48.9% lived in urban area, and health insurance coverage increased to 44.5%. Overall, low physical activity prevalence increased more than 2-fold from 18.2% (95% CI: 17.1–19.3) in 2007 to 39.6% (95% CI: 38.2–41.0) in 2014 (Table 2, Supplementary Table A2). Supplementary Figure A2 presents the prevalence of physical activity levels by sex and socioeconomic development.

**Table 2:** Prevalence of low physical activity and factor associated with physical activity levels.

**Variables**	**2007**		**2014**		**Factor associated with physical**	
	**Total**	**Physical activity**	**Total**	**Physical activity**	**activity levels (ref: high PA)** ^a^	
		**Low**		**Low**	**Low**	**Moderate**
	***n*** **(%)**	**% (95% CI)**	***n*** **(%)**	**% (95% CI)**	**AOR (95% CI)**	**AOR (95% CI)**
**Overall**	**5,936 (100)**	**18.2 (17.1–19.3)**	**5,936 (100)**	**39.6 (38.2–41.0)**		
**Sex**						
Male	2,650 (45.3)	13.9 (12.5–15.4)	2,649 (45.3)	35.1 (33.1–37.2)	1.00 (ref)	1.00 (ref)
Female	3,286 (54.7)	21.7 (20.1–23.3)	3,287 (54.7)	43.4 (41.4–45.3)	1.68 (1.41–1.98)^***^	1.65 (1.42–1.92)^***^
**Age**						
40–49 years	2,838 (48.4)	15.9 (14.4–17.4)	912 (16.2)	29.8 (26.5–33.2)	1.00 (ref)	1.00 (ref)
50–59 years	1,913 (31.8)	17.0 (15.2–18.9)	2,618 (43.4)	34.5 (32.5–36.6)	1.74 (1.53–1.98)^***^	1.32 (1.18–1.47)^***^
60–69 years	1,013 (17.2)	24.5 (21.6–27.5)	1,552 (26.1)	41.4 (38.7–44.2)	2.37 (2.02–2.78)^***^	1.45 (1.26–1.67)^***^
70–80 years	172 (2.7)	31.2 (23.3–39.1)	854 (14.2)	63.1 (59.3–66.8)	5.88 (4.63–7.46)^***^	2.04 (1.62–2.58)^***^
**Marital status**						
Not currently married	988 (16.3)	25.4 (22.3–28.4)	1,474 (24.1)	50.2 (47.3–53.1)	1.00 (ref)	1.00 (ref)
Currently married	4,948 (83.7)	16.8 (15.6–17.9)	4,462 (75.9)	36.3 (34.7–37.9)	0.88 (0.76–1.01)	0.97 (0.85–1.11)
**Ethnicity**						
Javanese	2,715 (52.3)	16.3 (14.7–17.8)	2,858 (55.7)	38.1 (36.2–40.1)	1.00 (ref)	1.00 (ref)
Sundanese	674 (15.9)	16.5 (13.6–19.3)	705 (16.5)	39.1 (35.4–42.9)	1.13 (0.95–1.35)	1.23 (1.05–1.43)^**^
Others	2,547 (31.8)	22.1 (20.3–24.0)	2,373 (27.8)	43.0 (40.6–45.3)	1.35 (1.17–1.55)^***^	1.25 (1.10–1.41)^***^
**Education**						
No education	2,888 (50.9)	16.6 (15.1–18.1)	3,018 (53.4)	42.1 (40.1–44.0)	1.00 (ref)	1.00 (ref)
Primary	1,514 (26.3)	17.1 (15.0–19.2)	1,395 (23.9)	35.9 (33.1–38.7)	1.10 (0.96–1.26)	1.13 (1.01–1.28)^**^
Junior high school	544 (8.4)	18.4 (14.8–22.0)	529 (8.1)	39.4 (34.7–44.1)	1.33 (1.09–1.62)^***^	1.28 (1.07–1.53)^***^
Senior high school	730 (10.7)	25.2 (21.6–28.7)	697 (10.3)	35.4 (31.4–39.4)	1.76 (1.45–2.13)^***^	1.68 (1.41–2.00)^***^
Tertiary	260 (3.8)	26.4 (20.0–32.9)	297 (4.4)	41.1 (34.4–47.9)	2.00 (1.50–2.67)^***^	1.88 (1.44–2.44)^***^
**Type of work**						
Unemployed	1,120 (18.0)	36.2 (33.0–39.4)	1,559 (25.4)	57.1 (54.3–59.9)	1.00 (ref)	1.00 (ref)
Casual worker	1,270 (22.6)	12.4 (10.4–14.5)	995 (17.9)	35.5 (32.1–38.9)	0.28 (0.24–0.34)^***^	0.49 (0.41–0.57)^***^
Self-employed	2,522 (43.0)	13.3 (11.8–14.7)	2,470 (42.0)	31.3 (29.2–33.3)	0.30 (0.25–0.35)^***^	0.54 (0.46–0.62)^***^
Government/private worker	1,024 (16.5)	19.1 (16.4–21.7)	912 (14.8)	38.4 (34.8–42.0)	0.42 (0.35–0.51)^***^	0.55 (0.46–0.66)^***^
**Residency**						
Rural	3,149 (62.5)	14.6 (13.3–15.9)	2,674 (51.1)	38.2 (36.2–40.3)	1.00 (ref)	1.00 (ref)
Urban	2,787 (37.5)	24.1 (22.3–26.0)	3,262 (48.9)	41.1 (39.2–43.1)	1.46 (1.30–1.64)^***^	1.23 (1.11–1.36)^***^
**Region of residency**						
Java-Bali	3,802 (76.5)	17.4 (16.1–18.7)	3,805 (76.5)	37.3 (35.6–39.0)	1.00 (ref)	1.00 (ref)
Sumatera	1,181 (15.5)	16.7 (14.5–18.8)	1,182 (15.6)	47.3 (44.3–50.3)	1.11 (0.96–1.28)	0.69 (0.61–0.79)^***^
Nusa Tenggara	403 (2.5)	18.4 (14.5–22.3)	403 (2.5)	31.4 (26.7–36.0)	0.76 (0.60–0.97)^**^	0.77 (0.63–0.95)^**^
Kalimantan	273 (1.8)	24.9 (19.7–30.0)	273 (1.8)	38.8 (33.0–44.7)	2.21 (1.66–2.94)^***^	1.92 (1.49–2.46)^***^
Sulawesi	277 (3.8)	36.5 (30.6–42.4)	273 (3.7)	62.3 (56.3–68.2)	3.48 (2.58–4.71)^***^	1.68 (1.26–2.24)^***^
**PCE**						
Q1	1,218 (23.1)	14.2 (12.1–16.3)	1,264 (23.8)	42.7 (39.7–45.8)	1.00 (ref)	1.00 (ref)
Q2	1,304 (23.2)	15.6 (13.4–17.8)	1,224 (21.3)	40.6 (37.5–43.8)	1.03 (0.88–1.20)	0.98 (0.85–1.13)
Q3	1,205 (20.2)	20.0 (17.5–22.5)	1,189 (20.1)	38.5 (35.4–41.7)	1.18 (1.00–1.39)^**^	1.15 (0.99–1.33)
Q4	1,188 (18.7)	19.9 (17.3–22.5)	1,141 (18.0)	35.8 (32.6–39.0)	1.09 (0.91–1.29)	1.13 (0.97–1.32)
Q5	1,021 (14.9)	23.6 (20.7–26.6)	1,118 (16.8)	39.4 (36.1–42.7)	1.41 (1.17–1.69)^***^	1.28 (1.08–1.52)^***^
**Insurance coverage**						
No	4,220 (73.1)	17.1 (15.8–18.4)	3,065 (55.5)	38.0 (36.1–40.0)	1.00 (ref)	1.00 (ref)
Yes	1,716 (26.9)	21.1 (18.9–23.2)	2,871 (44.5)	41.6 (39.5–43.7)	1.18 (1.06–1.32)^***^	0.99 (0.89–1.10)
**Tobaccu use (ref. non-smoker)**					
Non-smoker	3,705 (61.4)	20.5 (19.0–21.9)	3,573 (59.4)	41.7 (39.9–43.6)	1.00 (ref)	1.00 (ref)
Former smoker	217 (3.1)	20.1 (14.1–26.1)	513 (7.9)	45.0 (40.0–49.9)	1.25 (0.97–1.60)	1.17 (0.93–1.47)
Light smoker	534 (9.0)	15.6 (12.3–18.9)	562 (9.9)	37.5 (33.0–42.1)	0.93 (0.75–1.15)	0.84 (0.70–1.02)
Moderate	1,184 (21.6)	12.6 (10.5–14.6)	1,004 (18.2)	31.6 (28.4–34.8)	0.76 (0.63–0.92)^***^	0.78 (0.67–0.92)^***^
Heavy	296 (4.8)	17.5 (12.6–22.5)	284 (4.7)	39.5 (32.9–46.2)	1.06 (0.81–1.39)	0.90 (0.70–1.15)
**Food consumption last week (mean days)**					
Fruit	4.27 (4.17–4.38)	4.09 (3.86–4.33)	3.42 (3.30–3.55)	2.90 (2.74–3.06)	0.96 (0.94–0.97)^***^	0.98 (0.96–0.99)^***^
Vegetables	7.26 (7.16–7.37)	7.05 (6.84–7.26)	4.69 (4.56–4.81)	3.72 (3.55–3.88)	0.89 (0.88–0.91)^***^	0.95 (0.94–0.97)^***^
Meat	1.46 (1.40–1.51)	1.43 (1.32–1.53)	1.30 (1.24–1.35)	1.04 (0.96–1.11)	0.98 (0.94–1.01)	1.00 (0.97–1.03)
Fish	3.43 (3.34–3.51)	3.71 (3.54–3.87)	2.82 (2.73–2.92)	2.41 (2.29–2.53)	0.97 (0.95–0.99)^***^	1.03 (1.01–1.05)^***^
Dairy	1.14 (1.07–1.21)	1.26 (1.11–1.40)	0.95 (0.88–1.01)	0.85 (0.76–0.93)	1.01 (0.99–1.04)	1.01 (0.99–1.04)

Prevalence of low physical activity and factors associated with physical activity levels in Indonesia between 2007 and 2014 (*n* = 5,936). AOR, adjusted odds ratio; CI, confidence interval; PA, physical activity; PCE, per capita expenditure. Values are unweighted counts and weighted percentages. Significance:

** *P* < 0.05;

*** *P* < 0.01.

a AOR was estimated using multinomial logistic regression for panel data (reference: high physical activity).

### Factor associated with physical activity levels

The results of multinomial logistic regression are presented in Table 2. Being female, non-Javanese ethnicity, unemployed, and living in an urban area outside Java-Bali or Nusa Tenggara region were factors significantly associated with higher odds of having low or moderate physical activity (high PA was reference category). Our results indicate a significant association between lower physical activity and higher socioeconomic and education status. Respondents in the highest household expenditure were more likely to have low physical activity (AOR 1.41, 95% CI: 1.17–1.69) over high physical activity, compared with those in the lowest household expenditure quintile.

### Physical activity and health outcomes

Overall, compared with those with high physical activity, respondents with low physical activity had higher prevalence of overweight/obesity, hypertension, diabetes, and CVDs (Supplementary Table A3). We also observed increasing prevalence of CVD and the risk factors between 2007 and 2014. For example, the prevalence of hypertension and diabetes increased from 49.1 to 61.2% and from 3.0 to 6.5% among those with low PA, respectively.

In 2014, the prevalence of “high 10-year risk of CVD” (≥20% chance of developing a cardiovascular event over 10 years) was lowest in the high physical activity group (5.4%, 95% CI: 4.0–6.6) and the highest was in low physical activity group (14.8%, 95% CI: 13.1–16.4) (Supplementary Table A3). Both men and women with low physical activity were predicted to have higher 10-year CVD risk compared with higher physical activity groups.

**Figure 1:**
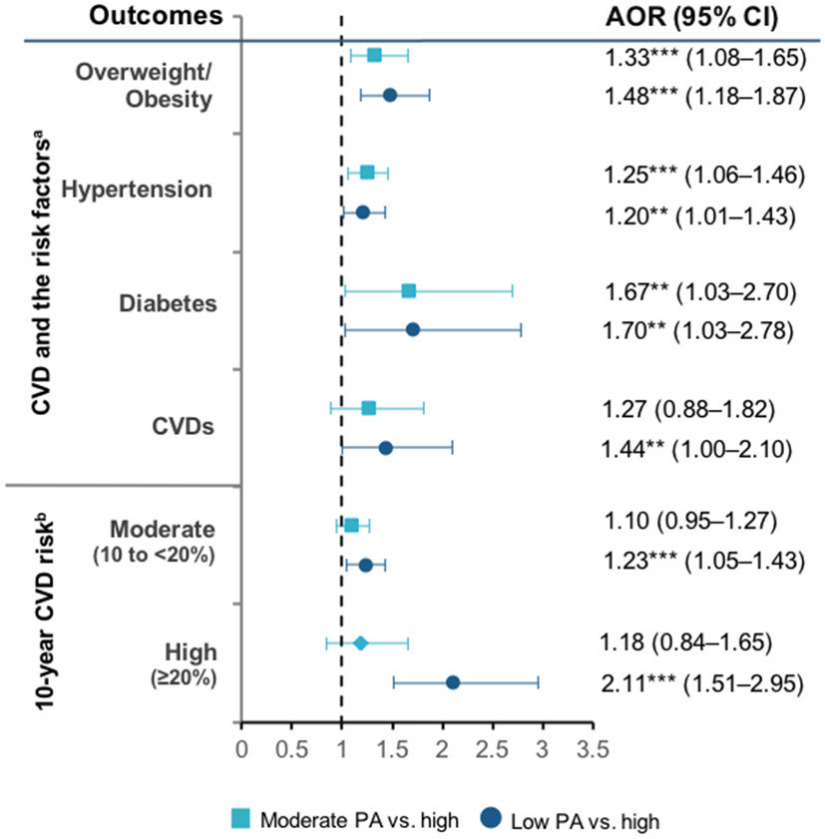
Physical activity levels and CVD, CVD risk factors, and WHO 10-year CVD risk. PA, physical activity. Ten-year CVD risk categories referred to the 2019 WHO CVD risk non-laboratory-based charts (Southeast Asia) that consider age, tobacco use, gender, systolic blood pressure, and BMI to derive the risk categories.

Figure 1 provides the AOR from the multilevel logistic regression to examine the association between physical activity and CVDs risk. Low physical activity was positively associated with higher likelihood of overweight/obesity (AOR 1.48, 95% CI: 1.18–1.87), hypertension (AOR 1.20, 95% CI: 1.01–1.43), and diabetes (AOR 1.70, 95% CI: 1.03–2.78), compared with high physical activity. The results also indicate that respondents with low physical activity had higher odds of having CVD (AOR 1.44, 95% CI: 1.00–2.10). In terms of 10-year CVD risk, low physical activity was also a significant predictor of moderate (AOR 1.23, 95% CI: 1.05–1.43) and high (AOR 2.11, 95% CI: 1.51–2.95) risk of CVD compared with high physical activity. The regression results are detailed in Supplementary Tables A4, A5.

### Physical activity, health service utilization, financial burden, and productivity loss

#### Health service utilization

**Figure 2:**
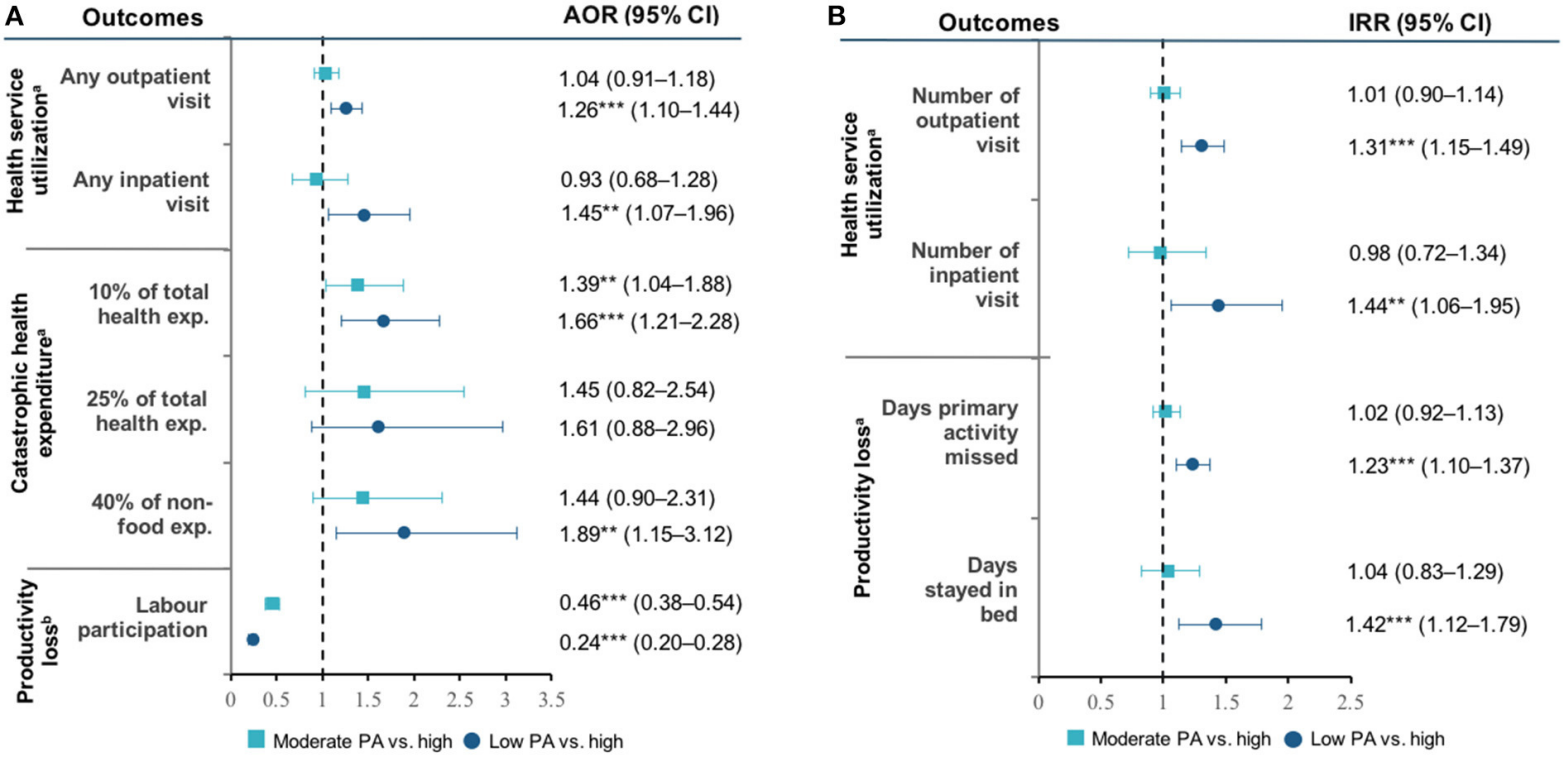
**(A,B)** Physical activity and health outcomes. PA, physical activity; CVDs, cardiovascular diseases; AOR, adjusted odds ratio. AOR was estimated using multilevel logistic regression model. ^a^Controlled by sex, age, marital status, ethnicity, education, type of work, residency, region of residency, PCE, insurance coverage, tobacco-use, and food-consumption. ^b^Controlled by marital status, ethnicity, education, type of work, residency, region of residency, PCE, insurance coverage, food-consumption. Significance: **P < 0.05; ***P < 0.01.

Respondents with low physical activity had higher rates of outpatient and inpatient visits compared with higher physical activity levels, both in 2007 and 2014 (Supplementary Table A3). Figure 2A shows that low physical activity increased the probability of using outpatient (AOR 1.26, 95% CI: 1.10–1.44) and inpatient care (AOR 1.45, 95% CI: 1.07–1.96) (Figure 2A). Among those with low physical activity, the incidence of outpatient (IRR 1.31, 95% 1.15–1.49) and inpatient visit (IRR 1.44, 95% 1.06–1.95) were also higher than those with high physical activity (Figure 2B). Having moderate physical activity did not significantly influence health service utilization compared to those with high physical activity. Detailed regression results are available in Supplementary Table A6.

#### Financial burden

The financial burden due to health service utilization was greater among those with low physical activity (Supplementary Table A3). In 2014, the mean OOPE for outpatient care incurred in the last 4 weeks increased from US$3.5 (95% CI: 2.3–4.6) in those with high physical activity to US$6.5 (95% CI: 2.5–10.4) in those with low physical activity. Similarly, the OOPE for inpatient care was 2-fold higher among those with low physical activity ($36.7, 95% CI: 21.9–51.6) compared with high physical activity ($15.8, 95% CI: 7.2–24.5).

Figure 2A presents the association between physical activity and the incidence of CHE. At 10% of total household expenditure threshold, those with low physical activity were more likely (AOR 1.66, 95% CI: 1.21–2.28) to experience CHE than those with high physical activity. Furthermore, using 40% of non-food expenditure threshold, having low physical activity increased the odds of experiencing CHE (AOR 1.89, 95% CI: 1.15–3.12) compared with high physical activity. We found no significant association between physical activity and CHE at 25% threshold. Detail regression results are available in Supplementary Table A7.

#### Work productivity

Respondents with low physical activity had the highest productivity loss. In 2014, among those aged 40–49 years, labor participation decreased from 93.1% (95% CI: 89.6–96.6) in those with high physical activity to 77.9% (95% CI: 71.6–84.2) in those with low physical activity (Supplementary Table A3). A similar finding was also found in older age groups. Figure 2B shows that those with low physical activity had a higher incidence rate of primary activity missed due to illness (IRR 1.23, 95% CI: 1.10–1.37) and days confined in bed (IRR 1.42, 95% CI: 1.12–1.79) compared with those with high physical activity. Detail regression results are available in Supplementary Table A8.

We reported the results of ICC in Supplementary Table 9. We found that the proportions of variance in all outcome variables generally appeared to be greater at the individual levels than at the household levels. The individual-level ICC was ranging from 12% (any outpatient visit) to 89% (overweight/obesity).

### Sensitivity analysis

To address the potential bias due to reverse causation, we excluded participants with 1+ADL in both waves 4 and 5 and repeated the analysis. This exclusion reduced the sample size to 3,424 observations. The results in sensitivity analysis are similar to those from our main analysis and suggest that they are robust (Supplementary Tables A10–A13).

## Discussion

### Principal findings

Our study presents a comprehensive panel data analysis on the association between PA levels and adverse health outcome, health care cost and productivity in Indonesia population. The prevalence of low physical activity in Indonesia increased from 18.2% in 2007 to 39.6% in 2014. Participants who were female, older and unemployed were more likely to have low PA. We also found that low PA was more prevalent among respondents living in urban area, were in the richest quintile and more educated. Low physical activity was associated with a higher prevalence of overweight, obesity, hypertension, diabetes, and risk of having CVDs. This study also highlights the strong association between PA and higher health service utilization for both ambulatory and inpatient care, greater risk of CHE, and poorer labor market participation.

Analyses of the socioeconomic gradient of physical activity in HICs tend to find a positive association where the wealthier were more physically active (24). In contrast, our study shows that wealthier and more educated groups tend to be less physically active, which echoed previous studies in Indonesia and other LMICs (25, 26). This may be due to occupational-related factors where higher-income and more educated population in LMICs are more likely to hold occupations that are less physically intense (27).

Our findings are aligned with earlier studies from both HICs and LMICs showing insufficient physical activity increases the odds of adverse health outcomes, healthcare use and costs (28, 29). A large-scale from 46 LMICs found that low physical activity was associated with most chronic conditions (30). Previous studies have also established the health benefits of physical activity in reducing the rates of all-cause mortality, NCDs, depression as well as certain types of cancer (31, 32). Further, this study also provides more robust evidence on the association between insufficient physical activity and productivity loss in LMICs (28).

### Policy implications

Improving physical activity is a key priority for many countries to improve NCD outcomes and prevent its associated costs. While WHO member states have agreed to reduce physical inactivity by 15% by 2030 (33), many LMICs are not making adequate progress to meet this target. This is often a result of funding barriers and limited technical capacity for developing a multi-sectoral interventions with multiple stakeholders (34). Indonesia implemented a National Action Plan on preventive and promotive measures for NCDs called Germans (healthy community movement) in 2015, which includes the promotion of physical activity (35). Some examples of program showing success include the urban-centered Indonesia SeGar. The program involved the provincial government setting policies and guidelines through the local related government institutions. Schools were also engaged and provided with exercise and sports equipment for physical education classes. Involving children and young adolescent is important in addressing the growing prevalence of overweight among Indonesian younger population and is a form of life-course perspective in improving physical activity (3). The program also engaged other stakeholders, including the private sector and the Indonesian Sports Medicine Doctor Association. Another successful physical activity intervention is an Active Park set with sports equipment, which has been shown to increase physical activity levels in adults in both LMICs and HICs (36–38), and can address the issue of an increasing obesogenic built environment.

Multi-stakeholder collaborations between the local primary healthcare with community organizations could help engage and educate the public to increase their physical activity levels. Government programs could play a role by training local health professionals within primary care settings to prescribe suitable physical activity interventions. Findings from this study can be utilized to identify groups of individuals with a higher likelihood of being physically inactive, such as females, older age groups, and those who live in urban areas, to recommend a training plan to improve their physical activity. In clinical settings, some progress has already been made in incorporating physical activity as part of treatment and prevention efforts. The American College of Sports Medicine (ACSM) has initiated Exercise is Medicine (EIM), which has been scaled up into a global initiative to make physical activity assessment and promotion a standard in clinical care (39). Through EIM, in Indonesia, physicians and health care providers are educated and trained by Indonesian Sports Medicine Doctors Association to design treatment plans that include physical activity and referring patients to qualified exercise professionals such as the Indonesia Physical Trainer Association.

These concerted efforts by different interested stakeholders highlight the importance of promotion of physical activity as integral to the prevention and treatment of chronic disease.

### Strengths and limitations

To the best of our knowledge, this is the first study in Indonesia that assess the adverse health outcome and costs associated with low physical activity among older population using population-based longitudinal dataset in Indonesia. The use of IPAQ-SF for older population is considered to have good test–retest reliability and adequate validity (40, 41). Our findings have several caveats. First, our regression analysis was to investigate the association rather than imply causation as the associations between physical activity and health status can be bi-directional. However, causal interpretation can be supported by the longitudinal study design and consistent dose-response relationship. We attempted to address the possible reverse causation by excluding respondents with disability in the sensitivity analysis, which yielded consistent findings. These findings need to be confirmed with further prospective longitudinal studies with longer term follow up. Second, respondents were reported to have difficulties in distinguishing moderate and vigorous activities in the IPAQ (40, 41). Furthermore, the self-reported questionnaire used to assess physical activity and outcome variables may have been subjected to reporting bias, leading to overestimation high physical activity level. The measurements of outcome variables using self-reported measures may also be prone to recall and ascertainment bias. For instance, the true prevalence of CVD and risk factors may have been underestimated among participants with limited health literacy and access to healthcare services. Third, physical activity was measured in the week prior to the interview, and thus, the measurement might not have accurately demonstrated individuals' seasonal activity, e.g., for those with agricultural occupations or self-employed. Fourth, the absence of certain biomarker measurements in IFLS, e.g., total and blood sugar level, may result in an underestimation of the CVD risk prediction. However, non-laboratory measurements are still recommended in settings where such data is not available. Fifth, the IFLS sample did not include the eastern provinces of Indonesia, which are mostly remote and have limited healthcare resources. Therefore, further exploration of the burden of low physical activity is warranted in these remaining provinces. Lastly, since the IFLS-5 was carried out between 2014 and 2015, this study may not capture the current prevalence of low physical activity in Indonesia. Moreover, yet even with this limitation, IFLS is still preferred compared with other national cross-sectional datasets (e.g., Indonesia Basic Health Research/RISKESDAS), considering the longitudinal design of the survey.

## Conclusions

Low physical activity is associated with higher prevalence of CVDs and the risk factors, and higher costs for the individuals and wider economy in Indonesia. This study underscores the importance of prioritizing interventions to improve PA level within the population, to reduce the risk and prevalence of CVDs, the associated healthcare costs and the indirect costs, related to loss of work productivity. Policies and programs should be tailored to provide supportive environments that enable the community to increase PA in a sustainable and collaborative manner.

## Data availability statement

Publicly available datasets were analyzed in this study. The datasets supporting the conclusions of this article are available after registration in: https://www.rand.org/well-being/social-and-behavioral-policy/data/FLS/IFLS/access.html.

## Ethics statement

The IFLS has been approved by ethics review boards at RAND Corporation and Gadjah Mada University in Indonesia. Written informed consent was sought from all respondents prior to data collection. As this study used publicly available datasets that contain no personal identification of the respondents, no further ethical approval was sought.

## Author contributions

KA, TM, MZ, and JL conceived and designed the study. KA and MZ did the initial analysis. TM, TH, and JL supervised data analysis. NN, AL, BM, CM, TH, EH, and YC contributed to interpretation of data. KA, TM, MZ, and SH wrote the first draft of the paper. NN, AL, BM, CM, TH, YC, and JL critically revised the initial draft. EH assisted in drafting the discussion section and proofread all section. All authors reviewed and approved the final version of the paper submitted for publication.
