# Supplementary material for: Low physical activity is associated with adverse health outcome and higher costs in Indonesia: A national panel study
Source: Front Cardiovasc Med. 2022 Dec 16;9:972461. doi: 10.3389/fcvm.2022.972461 (PMC9800782; doi:10.3389/fcvm.2022.972461)
Supplement: Supplementary file 1 [file Table_1.DOCX]

**Low physical activity is associated with adverse health outcome and higher costs in Indonesia: a national panel study**

**SUPPLEMENTAL MATERIALS**

**Figure A1. Sample flowchart**

**Figure A2. Prevalence of physical activity, by sex and socioeconomic development**

**Notes:**

PA: Physical activity

Prevalence was age- and sex-standardised, using the age distribution of Indonesian population, as per the 2010 Indonesian Population Census.

**Figure A3. Physical activity levels and WHO 10-year CVD risk**

**Notes:**

PA: Physical activity

10-year CVD risk categories referred to the 2019 WHO CVD risk non-laboratory-based charts (Southeast Asia) that consider age, tobacco use, gender, systolic blood pressure, and BMI to derive the risk categories.

**Table A1. List of variables for 2007 & 2014 IFLS analysis**

| **Variables** | **Type** | **Measurement** | **Source of measurement** |
| --- | --- | --- | --- |
| **Dependent variables:** | | | |
| 1. Adverse health outcomes | | | |
| Overweight/ obesity  Hypertension  Diabetes  CVDs  10-year CVD risk | Binary  Binary  Binary  Binary  Categorical ordinal | 0. No  1. Yes (≥23 kg/m^2^)  0. No  1. Yes  0. No  1. Yes  0. No  1. Yes   1. <5% 2. 5% – <10% (low) 3. 10% – <20% (moderate) 4. ≥20% (high) | US04: height (cm)  US06: weight (kg)  CD05: Have a doctor/paramedic/nurse/midwife ever told you that you had hypertension?  US07a–c: blood pressure measurement (systolic blood pressure of ≥140 mmHg and/or diastolic blood pressure of ≥90 mmHg)  USE18aB: Are you taking medicine for High Blood Pressure?  CD05: Have a doctor/paramedic/nurse/midwife ever told you that you had diabetes?  USE18aB: Are you taking medicine for Diabetes?  CD05: Have a doctor/paramedic/nurse/midwife ever told you that you had Heart attack,  coronary heart disease, angina, or other heart problems *or* stroke?  Measurement was based on 2019 WHO CVD risk chart, by considering sex, age, tobacco use, BMI, and systolic blood pressure. |
| 1. Health service use | | | |
| Outpatient care | Binary  Numerical | 0. No  1. Yes  Number of days | RJ00: In the last 4 weeks have you visited a public hospital-*puskesmas*-private hospital-clinic-health worker or doctor’s practice or been visited by a health worker or doctor?  RJ02: How many times did you visit / been visited by [...] duringthe last 4 weeks? |
| Inpatient care | Binary  Numerical | 0. No  1. Yes  Number of days | RN00: During the past 12 months have you ever received patient care at a hospital-*puskesmas*-clinic-or  other?  RN02: How many times have you received inpatient care at […] during the past 12  months? |
| 1. Productivity loss | | | |
| Labour participation | Binary  Numerical | 0. No  1. Yes  Number of days | TK06a: Did you work/try to work/help to earn income for pay for at least 1 hour during the past week? |
| Activity missed due to poor health | Numerical | Number of days | KK02a: During the last 4 weeks-  how many days of  your primary daily activities did you miss due to  poor health? |
| Stayed in bed | Numerical | Number of days | KK02b: In the last 4 weeks-how many days have you stayed in bed due to poor health? |
| 1. Financial burden | | | |
| OOPE of outpatient care | Numerical | USD | RJ02b: How much did you pay out of pocket for outpatient care at […] during the past 4 weeks? |
| OOPE of  inpatient care | Numerical | USD | RN02b: How much did you pay out of pocket for inpatient care at […] during the past 12 months? |
| Catastrophic health expenditure | Binary | 1. No 2. Yes | Book KS:  "How much money spent by all household members for medical costs during the past year?" |
| **Main independent variable** | | | |
| Physical activity | Categorical nominal | 1. Low 2. Moderate 3. High | KK02m: During the last 7 days, did you do any [vigorous/moderate/walking] for at least 10 minutes continuously? (yes/no)  KK02n: How much time did you usually spend doing [vigorous/moderate/walking] on one of those days? (recorded in minutes)  KK02o: During the last 7 days, on how many days did you do [vigorous/moderate/walking]? (recorded in days) |
| Vigorous activities: heavy lifting, digging, plowing, aerobics, fast bicycling, cycling with loads.  Moderate activities: carrying light loads, bicycling at a regular pace, or mopping the floor.  **Computation of MET-minutes (IPAQ)**   - Walking MET-minutes/week = 3.3*walking minutes*walking days - Moderate MET-minutes/week = 4.0*moderate-intensity activity minutes*moderate-intensity - Vigorous MET-minutes/week at work= 8.0*vigorous-intensity activity minutes*vigorous-intensity - Total Work MET-minutes/week =sum of Walking + Moderate + Vigorous MET-minutes/week scores   **Categorisation of physical activity levels (IPAQ)**  Category 1 Low: Individuals who not meet criteria for Categories 2 or 3 are considered ‘low’.  Category 2 Moderate: Individuals meeting at least one of the below criteria.   1. 3 or more days of vigorous-intensity activity of at least 20 minutes per day, or 2. 5 or more days of moderate-intensity activity and/or walking of at least 30 minutes per day, or 3. 5 or more days of any combination of walking, moderate-intensity or vigorous intensity activities achieving a minimum Total physical activity of at least 600 MET-minutes/week.   Category 3 High: Individuals meeting at least one of the below criteria.   1. vigorous-intensity activity on at least 3 days achieving a minimum Total physical activity of at least 1500 MET-minutes/week 2. 7 or more days of any combination of walking, moderate-intensity or vigorous-intensity activities achieving a minimum Total physical activity of at least 3000 MET-minutes/week. | | | |
| **Covariates** | | | |
| Age (in years) | Categorical ordinal | 1. 40-49 years 2. 50-59 years 3. 60-69 years 4. 70-79 years 5. 80+ | Book IIIA:  Age: How old are you? |
| Sex | Binary | 1. Male 2. Female | Book IIIA:  Sex: (identified by interviewers) |
| Ethnicity | Categorical nominal | 1. Javanese 2. Sundanese 3. Others |  |
| Marital status | Binary | 1. Unmarried/Divorce 2. Married or living together | Book IIIA  HR00b: Are you currently married? |
| Education | Categorical ordinal | 1. None 2. Elementary school 3. Junior high school 4. High school 5. Tertiary | Book IIIA:  DL06: What is the highest education level  attended?  DL07: What is the highest grade completed at school. |
| Occupation | Categorical nominal | 1. None 2. Casual worker 3. Self-employed 4. Government/private worker | Book IIIA:  TK06a: Did you work/try to work/help to  earn income for pay for at least  1 hour during the past week?  TK15: Which category best describes the work you did in your last job? |
| Residency | Binary | 1. Rural 2. Urban | Book T-2:  SC06: (identified by interviewers) |
| Region | Categorical nominal | 1. Java-Bali 2. Sumatra 3. Nusa Tenggara 4. Kalimantan 5. Sulawesi | Book T-2:  SC01: province (identified by interviewers) |
| Per capita expenditure (PCE) | Categorical ordinal | 1. Q1 (lowest) 2. Q2 3. Q3 4. Q4 5. Q5 (highest) | Book KS |
| Health insurance status | Binary | 1. Uninsured (Not covered by any insurance) 2. Insured | Book IIIB:  AK01: Are you the policy holder/primary beneficiary of health benefits-health insurance? |
| Residency | Binary | 1. Rural 2. Urban | Book T-2:  SC06: (identified by interviewers) |
| Region | Categorical nominal | 1. Java-Bali 2. Sumatra 3. Nusa Tenggara 4. Kalimantan 5. Sulawesi | Book T-2:  SC01: province (identified by interviewers) |
| Tobacco use | Categorical nominal | 1. Non-user 2. Former user 3. Light user 4. Moderate user 5. Heavy user | KM01a: Have you ever chewed tobacco, smoked a pipe, smoked self-rolled cigarettes, or smoked cigarettes/cigars?  KM04: Do you still have the habit or have you totally quit?  KM08: In one day about how many cigars/cigarettes did you consume now/before totally quitting?  KM06: In one week how many ounces (100 grams) did/do you consume now/before totally quitting of chewing tobacco and smoking pipe? |
| Food consumption | Numerical | Fruit  Vegetable  Meat  Dairy products  Fish | FMTYPE: type of food you usually eat  FM02: In the last week, did you eat any [list of food]?  FM03: How many days in a week did you eat [list of food] in the last week? |
| Body mass index | Numerical |  | US04: height  US06: weight (kg) |
| Number of NCDs | Categorical ordinal | 1. No 2. 1 NCD 3. 2 NCDs 4. 3+ NCDs | Book IIIB:  CD06a – CD06r: Have a  doctor/paramedic/nurse/ midwife ever told you that you had [list of chronic diseases] |
| **Measurement of limitation of** **limitation in activities of daily living (ADL) sensitivity analysis:**  Respondents were categorized as having limitation if they answered “unable to do it” in at least 1 (1+ADL) of the 15 selected physical functioning measures (below) that were constantly measured in IFLS-4 and IFLS-5:   1. KK03a. To carry a heavy load (like a pail of water) for 20 meters 2. KK03b. To sweep the house floor yard 3. KK03c. To walk for 5 kilometers 4. KK03d. To draw a pail of water from a well 5. KK03e. To bow, squat, kneel 6. KK03g. To stand up from sitting position in a chair without help 7. KK03i. To stand up from sitting on the floor without help 8. KK03j. To walk for 1 kilometer 9. KK03l. To walk across the room   Activities of daily living (ADL)   1. KK03f. To dress without help 2. KK03k. To get out of bed 3. KK03m. To bathe   Instrumental activities of daily living (IADL)   1. KK03n. To shop for personal needs 2. KK03o. To prepare hot meals (preparing ingredients, cooking, and serving food) 3. KK03p. To take medicine (taking right portion right on time) | | | |

**Table A2. Prevalence of physical activity level across different sociodemographic and economic groups**

| **Variables** | | **2007** | | | |  | **2014** | | | |
| --- | --- | --- | --- | --- | --- | --- | --- | --- | --- | --- |
|  |  | **Total** | **Physical activity** | | |  | **Total** | **Physical activity** | | |
|  |  |  | **Insufficient** | **Moderate** | **High** |  |  | **Insufficient** | **Moderate** | **High** |
|  |  | n (%) | % (95% CI) | % (95% CI) | % (95% CI) |  | n (%) | % (95% CI) | % (95% CI) | % (95% CI) |
| **Overall** | | **5936 (100)** | **18.2 (17.1–19.3)** | **34.2 (32.8–35.6)** | **47.6 (46.1–49.1)** |  | **5936 (100)** | **39.6 (38.2–41.0)** | **32.3 (31.0–33.8)** | **28.0 (26.8–29.3)** |
| Sex |  |  |  |  |  |  |  |  |  |  |
|  | Male | 2650 (45.3) | 13.9 (12.5–15.4) | 29.1 (27.2–31.1) | 56.9 (54.8–59.1) |  | 2650 (45.3) | 35.1 (33.1–37.2) | 28.5 (26.6–30.5) | 36.3 (34.2–38.5) |
|  | Female | 3286 (54.7) | 21.7 (20.1–23.3) | 38.4 (36.5–40.3) | 39.9 (38.0–41.8) |  | 3286 (54.7) | 43.4 (41.4–45.3) | 35.6 (33.7–37.5) | 21.0 (19.4–22.6) |
| Age | |  |  |  |  |  |  |  |  |  |
|  | 40-49 years | 2838 (48.4) | 15.9 (14.4–17.4) | 33.5 (31.5–35.5) | 50.6 (48.5–52.7) |  | 912 (16.2) | 29.8 (26.5–33.2) | 36.7 (33.2–40.3) | 33.4 (29.9–36.9) |
|  | 50–59 years | 1913 (31.8) | 17.0 (15.2–18.9) | 35.6 (33.1–38.0) | 47.4 (44.8–49.9) |  | 2618 (43.4) | 34.5 (32.5–36.6) | 34.3 (32.2–36.3) | 31.2 (29.2–33.3) |
|  | 60–69 years | 1013 (17.2) | 24.5 (21.6–27.5) | 32.8 (29.4–36.1) | 42.7 (39.2–46.2) |  | 1552 (26.1) | 41.4 (38.7–44.2) | 32.4 (29.7–35.0) | 26.2 (23.7–28.7) |
|  | 70–80 years | 172 (2.7) | 31.2 (23.3–39.1) | 40.5 (31.9–49.1) | 28.3 (20.5–36.1) |  | 854 (14.2) | 63.1 (59.3–66.8) | 21.8 (18.6–24.9) | 15.2 (12.3–18.0) |
| Marital status | |  |  |  |  |  |  |  |  |  |
|  | Not currently married | 988 (16.3) | 25.4 (22.3–28.4) | 37.0 (33.6–40.4) | 37.6 (34.2–41.1) |  | 1474 (24.1) | 50.2 (47.3–53.1) | 30.7 (28.0–33.4) | 19.1 (16.8–21.4) |
|  | Currently married | 4948 (83.7) | 16.8 (15.6–17.9) | 33.7 (32.2–35.2) | 49.6 (48.0–51.2) |  | 4462 (75.9) | 36.3 (34.7–37.9) | 32.9 (31.4–34.5) | 30.8 (29.2–32.4) |
| Ethnicity | |  |  |  |  |  |  |  |  |  |
|  | Javanese | 2715 (52.3) | 16.3 (14.7–17.8) | 33.3 (31.3–35.2) | 50.5 (48.4–52.5) |  | 2858 (55.7) | 38.1 (36.2–40.1) | 31.0 (29.1–32.8) | 30.9 (29.0–32.8) |
|  | Sundanese | 674 (15.9) | 16.5 (13.6–19.3) | 36.3 (32.5–40.1) | 47.2 (43.2–51.2) |  | 705 (16.5) | 39.1 (35.4–42.9) | 35.9 (32.2–39.6) | 25.0 (21.5–28.4) |
|  | Others | 2547 (31.8) | 22.1 (20.3–24.0) | 34.7 (32.5–36.9) | 43.2 (40.9–45.4) |  | 2373 (27.8) | 43.0 (40.6–45.3) | 33.1 (30.9–35.4) | 23.9 (21.9–26.0) |
| Education | |  |  |  |  |  |  |  |  |  |
|  | No education | 2888 (50.9) | 16.6 (15.1–18.1) | 33.4 (31.5–35.4) | 50.0 (47.9–52.0) |  | 3018 (53.4) | 42.1 (40.1–44.0) | 29.6 (27.8–31.5) | 28.3 (26.5–30.2) |
|  | Primary | 1514 (26.3) | 17.1 (15.0–19.2) | 31.8 (29.1–34.4) | 51.2 (48.3–54.0) |  | 1395 (23.9) | 35.9 (33.1–38.7) | 34.6 (31.8–37.5) | 29.5 (26.7–32.2) |
|  | Junior high school | 544 (8.4) | 18.4 (14.8–22.0) | 34.8 (30.2–39.3) | 46.8 (42.1–51.6) |  | 529 (8.1) | 39.4 (34.7–44.1) | 34.7 (30.1–39.3) | 26.0 (21.6–30.3) |
|  | Senior high school | 730 (10.7) | 25.2 (21.6–28.7) | 40.9 (36.7–45.0) | 34.0 (30.0–37.9) |  | 697 (10.3) | 35.4 (31.4–39.4) | 37.3 (33.2–41.4) | 27.3 (23.4–31.2) |
|  | Tertiary | 260 (3.8) | 26.4 (20.0–32.9) | 42.0 (34.7–49.2) | 31.6 (24.8–38.3) |  | 297 (4.4) | 41.1 (34.4–47.9) | 38.1 (31.4–44.9) | 20.7 (15.4–26.0) |
| Type of work | |  |  |  |  |  |  |  |  |  |
|  | Unemployed | 1120 (18.0) | 36.2 (33.0–39.4) | 38.1 (34.9–41.3) | 25.7 (22.8–28.6) |  | 1559 (25.4) | 57.1 (54.3–59.9) | 31.7 (29.1–34.3) | 11.2 (9.4–12.9) |
|  | Casual worker | 1270 (22.6) | 12.4 (10.4–14.5) | 33.9 (30.9–36.9) | 53.7 (50.5–56.8) |  | 995 (17.9) | 35.5 (32.1–38.9) | 31.5 (28.2–34.8) | 33.0 (29.6–36.3) |
|  | Self-employed | 2522 (43.0) | 13.3 (11.8–14.7) | 32.3 (30.2–34.4) | 54.4 (52.2–56.6) |  | 2470 (42.0) | 31.3 (29.2–33.3) | 33.5 (31.4–35.6) | 35.2 (33.1–37.4) |
|  | Government/private worker | 1024 (16.5) | 19.1 (16.4–21.7) | 35.4 (32.0–38.8) | 45.5 (42.0–49.0) |  | 912 (14.8) | 38.4 (34.8–42.0) | 31.5 (28.0–34.9) | 30.1 (26.7–33.6) |
| Residency | |  |  |  |  |  |  |  |  |  |
|  | Rural | 3149 (62.5) | 14.6 (13.3–15.9) | 33.1 (31.3–35.0) | 52.3 (50.3–54.2) |  | 2674 (51.1) | 38.2 (36.2–40.3) | 29.8 (27.9–31.7) | 32.0 (30.0–34.0) |
|  | Urban | 2787 (37.5) | 24.1 (22.3–26.0) | 36.0 (33.9–38.1) | 39.9 (37.8–42.0) |  | 3262 (48.9) | 41.1 (39.2–43.1) | 35.1 (33.2–37.0) | 23.8 (22.0–25.5) |
| Region of residency | |  |  |  |  |  |  |  |  |  |
|  | Java-Bali | 3802 (76.5) | 17.4 (16.1–18.7) | 36.3 (34.6–38.0) | 46.3 (44.6–48.1) |  | 3805 (76.5) | 37.3 (35.6–39.0) | 33.0 (31.4–34.7) | 29.7 (28.1–31.4) |
|  | Sumatera | 1181 (15.5) | 16.7 (14.5–18.8) | 22.5 (20.1–24.9) | 60.8 (58.0–63.7) |  | 1182 (15.6) | 47.3 (44.3–50.3) | 29.1 (26.4–31.8) | 23.6 (21.1–26.2) |
|  | Nusa Tenggara | 403 (2.5) | 18.4 (14.5–22.3) | 27.3 (22.9–31.7) | 54.3 (49.3–59.3) |  | 403 (2.5) | 31.4 (26.7–36.0) | 37.8 (32.9–42.6) | 30.9 (26.3–35.5) |
|  | Kalimantan | 273 (1.8) | 24.9 (19.7–30.0) | 46.7 (40.6–52.8) | 28.4 (22.9–34.0) |  | 273 (1.8) | 38.8 (33.0–44.7) | 40.0 (34.1–45.9) | 21.2 (16.3–26.1) |
|  | Sulawesi | 277 (3.8) | 36.5 (30.6–42.4) | 39.6 (33.6–45.6) | 23.9 (18.7–29.1) |  | 273 (3.7) | 62.3 (56.3–68.2) | 26.4 (21.0–31.7) | 11.4 (7.5–15.2) |
| PCE | |  |  |  |  |  |  |  |  |  |
|  | Q1 | 1218 (23.1) | 14.2 (12.1–16.3) | 33.2 (30.2–36.1) | 52.6 (49.5–55.7) |  | 1264 (23.8) | 42.7 (39.7–45.8) | 29.5 (26.7–32.3) | 27.7 (25.0–30.5) |
|  | Q2 | 1304 (23.2) | 15.6 (13.4–17.8) | 31.6 (28.8–34.5) | 52.8 (49.7–55.8) |  | 1224 (21.3) | 40.6 (37.5–43.8) | 30.2 (27.3–33.2) | 29.1 (26.2–32.1) |
|  | Q3 | 1205 (20.2) | 20.0 (17.5–22.5) | 35.3 (32.2–38.4) | 44.7 (41.5–47.9) |  | 1189 (20.1) | 38.5 (35.4–41.7) | 31.8 (28.8–34.8) | 29.6 (26.6–32.7) |
|  | Q4 | 1188 (18.7) | 19.9 (17.3–22.5) | 34.3 (31.2–37.4) | 45.8 (42.6–49.1) |  | 1141 (18.0) | 35.8 (32.6–39.0) | 36.7 (33.4–39.9) | 27.6 (24.5–30.6) |
|  | Q5 | 1021 (14.9) | 23.6 (20.7–26.6) | 38.3 (34.8–41.7) | 38.1 (34.6–41.6) |  | 1118 (16.8) | 39.4 (36.1–42.7) | 35.3 (32.0–38.5) | 25.3 (22.3–28.3) |
| Insurance coverage | |  |  |  |  |  |  |  |  |  |
|  | No | 4220 (73.1) | 17.1 (15.8–18.4) | 34.6 (33.0–36.3) | 48.3 (46.6–50.0) |  | 3065 (55.5) | 38.0 (36.1–40.0) | 32.8 (30.9–34.6) | 29.2 (27.4–31.0) |
|  | Yes | 1716 (26.9) | 21.1 (18.9–23.2) | 33.1 (30.5–35.7) | 45.8 (43.1–48.6) |  | 2871 (44.5) | 41.6 (39.5–43.7) | 31.9 (30.0–33.9) | 26.4 (24.5–28.3) |
| Tobacco use | |  |  |  |  |  |  |  |  |  |
|  | Non-smokers | 3705 (61.4) | 20.5 (19.0–21.9) | 37.8 (36.0–39.6) | 41.7 (39.9–43.5) |  | 3573 (59.4) | 41.7 (39.9–43.6) | 35.7 (33.9–37.5) | 22.5 (20.9–24.1) |
|  | Former smokers | 217 (3.1) | 20.1 (14.1–26.1) | 35.3 (28.0–42.6) | 44.5 (36.9–52.2) |  | 513 (7.9) | 45.0 (40.0–49.9) | 28.8 (24.3–33.2) | 26.3 (21.7–30.9) |
|  | Light smokers | 534 (9.0) | 15.6 (12.3–18.9) | 30.5 (26.1–34.9) | 53.9 (49.2–58.7) |  | 562 (9.9) | 37.5 (33.0–42.1) | 27.1 (23.0–31.3) | 35.3 (30.8–39.8) |
|  | Moderate smokers | 1184 (21.6) | 12.6 (10.5–14.6) | 26.6 (23.7–29.5) | 60.8 (57.7–64.0) |  | 1004 (18.2) | 31.6 (28.4–34.8) | 27.3 (24.2–30.3) | 41.1 (37.7–44.6) |
|  | Heavy smokers | 296 (4.8) | 17.5 (12.6–22.5) | 28.4 (22.4–34.4) | 54.1 (47.5–60.6) |  | 284 (4.7) | 39.5 (32.9–46.2) | 27.0 (21.3–32.7) | 33.5 (27.1–39.9) |
| Food consumption last week (mean days) | | |  |  |  |  |  |  |  |  |
|  | Fruit | 4.27 (4.17–4.38) | 4.09 (3.86–4.33) | 4.28 (4.10–4.45) | 4.34 (4.19–4.49) |  | 3.42 (3.30–3.55) | 2.90 (2.74–3.06) | 3.63 (3.45–3.81) | 3.45 (3.24–3.65) |
|  | Vegetables | 7.26 (7.155–7.37) | 7.05 (6.84–7.26) | 7.09 (6.93–7.24) | 7.35 (7.22–7.48) |  | 4.69 (4.56–4.81) | 3.72 (3.55–3.88) | 4.90 (4.72–5.08) | 4.88 (4.68–5.08) |
|  | Meat | 1.46 (1.40–1.51) | 1.43 (1.32–1.53) | 1.47 (1.39–1.55) | 1.33 (1.27–1.40) |  | 1.30 (1.24–1.35) | 1.04 (0.96–1.11) | 1.31 (1.23–1.40) | 1.31 (1.21–1.40) |
|  | Fish | 3.43 (3.34–3.51) | 3.71 (3.54–3.87) | 3.61 (3.49–3.73) | 3.33 (3.23–3.43) |  | 2.82 (2.73–2.92) | 2.41 (2.29–2.53) | 2.98 (2.85–3.11) | 3.06 (2.91–3.21) |
|  | Dairy | 1.14 (1.07–1.21) | 1.26 (1.11–1.40) | 1.22 (1.11–1.33) | 0.95 (0.87–1.03) |  | 0.95 (0.88–1.01) | 0.85 (0.76–0.93) | 0.96 (0.86–1.06) | 0.94 (0.83–1.05) |
| **Notes:** | |  |  |  |  |  |  |  |  |  |
| PCE: Per capita expenditure | |  |  |  |  |  |  |  |  |  |
| Values are unweighted counts and weighted percentages | | | |  |  |  |  |  |  |  |

**Table A3. Descriptive summary of health service use, financial burden, and productivity outcome, by physical activity**

| **Outcomes** | | **Physical activity** | | | **Physical activity** | | |
| --- | --- | --- | --- | --- | --- | --- | --- |
|  |  | **2007** | | | **2014** | | |
|  |  | Low | Moderate | High | Low | Moderate | High |
| **2007** | |  |  |  |  |  |  |
| **Overall, N (%)** | | 1042 (15.1) | 2258 (32.7) | 3604 (52.2) | 2679 (38.8) | 1948 (28.2) | 2276 (33.0) |
| **CVDs and risk factors for CVDs** | | |  |  |  |  |  |
|  | Overweight & obesity | 47.1 (43.8–50.4) | 45.2 (42.7–47.7) | 37.2 (35.2–39.2) | 46.7 (44.4–48.9) | 54.0 (51.5–56.5) | 40.3 (37.5–43.0) |
|  | Hypertension | 49.1 (45.8–52.3) | 46.6 (44.1–49.1) | 40.5 (38.4–42.5) | 61.2 (59.0–63.4) | 56.3 (53.8–58.9) | 49.9 (47.1–52.7) |
|  | Diabetes | 3.0 (2.0–4.1) | 2.1 (1.4–2.8) | 11.1 (0.7–1.5) | 6.5 (5.4–7.6) | 6.4 (5.2–7.6) | 3.3 (2.4–4.3) |
|  | CVDs | 2.6 (1.6–3.6) | 2.1 (1.4–2.8) | 1.4 (0.9–1.9) | 5.9 (4.9–7.0) | 4.9 (3.9–6.0) | 3.0 (2.1–4.0) |
|  | 10-year CVD risk |  |  |  |  |  |  |
|  | Low (5%–<10%) | 31.5 (28.4–34.6) | 32.8 (30.4–35.1) | 34.7 (32.6–36.7) | 32.4 (30.2–34.5) | 40.5 (38.0–43.0) | 43.7 (40.9–46.5) |
|  | Moderate (10%–<20%) | 22.1 (19.3–24.9) | 17.2 (15.3–19.1) | 15.4 (13.8–16.9) | 35.4 (33.2–37.6) | 29.7 (27.3–32.0) | 28.3 (25.8–30.8) |
|  | High (≥20%) | 3.62 (2.40–4.83) | 2.75 (1.90–3.56) | 2.53 (1.80–3.22) | 14.8 (13.1–16.4) | 6.43 (5.20–7.67) | 5.36 (4.00–6.65) |
| **Health service use** | |  |  |  |  |  |  |
|  | Outpatient^a)^ |  |  |  |  |  |  |
|  | Any visits (%, 95% CI) | 19.6 (17.0–22.2) | 17.7 (15.8–19.6) | 12.3 (11.0–13.7) | 25.1 (23.1–27.1) | 20.9 (18.8–22.9) | 19.7 (17.4–21.9) |
|  | Number of visits (mean, 95% CI) | 0.36 (0.28–0.44) | 0.28 (0.23–0.33) | 0.20 (0.17–0.23) | 0.52 (0.47–0.58) | 0.40 (0.34–0.47) | 0.36 (0.30–0.41) |
|  | Inpatient^b)^ |  |  |  |  |  |  |
|  | Any visits (%, 95% CI) | 3.0 (1.9–4.1) | 1.2 (0.7–1.6) | 1.4 (0.9–1.9) | 5.7 (4.6–6.7) | 4.1 (3.1–5.1) | 2.9 (2.0–3.8) |
|  | Number of visits (mean, 95% CI) | 0.03 (0.02–0.05) | 0.01 (0.01–0.02) | 0.01 (0.01–0.02) | 0.08 (0.06–0.10) | 0.06 (0.04–0.07) | 0.03 (0.02–0.05) |
| **Financial burden** | |  |  |  |  |  |  |
|  | OOPE for outpatient care (mean, 95% CI)^a,c)^ | 2.48 (1.59–3.38) | 1.66 (1.23–2.09) | 1.08 (0.82–1.33) | 6.47 (2.52–10.41) | 4.83 (3.58–6.08) | 3.47 (2.31–4.64) |
|  | OOPE for inpatient care (mean, 95% CI)^b,c)^ | 18.0 (7.9–28.2) | 7.9 (0.73–15.0) | 4.6 (1.5–7.7) | 36.7 (21.9–51.6) | 25.8 (14.9–36.7) | 15.8 (7.2–24.5) |
|  | Catastrophic health expenditure (%, 95% CI) |  |  |  |  |  |  |
|  | >10% of total household expenditure | 5.1 (3.7–6.5) | 5.1 (4.0–6.2) | 3.4 (2.6–4.2) | 7.2 (6.0–8.4) | 5.9 (4.7–7.0) | 4.8 (3.6–6.0) |
|  | >25% of total household expenditure | 1.7 (0.9–2.5) | 1.6 (0.9–2.2) | 1.1 (0.6–1.5) | 1.5 (1.0–2.1) | 1.5 (0.9–2.1) | 0.8 (0.3–1.3) |
|  | >40% of total non-food expenditure | 2.4 (1.4–3.4) | 2.3 (1.5–3.1) | 1.5 (0.9–2.0) | 2.5 (1.8–3.2) | 2.0 (1.3–2.7) | 1.5 (0.8–2.2) |
| **Productivity loss** | |  |  |  |  |  |  |
|  | Labour participation (%, 95% CI)^d)^ |  |  |  |  |  |  |
|  | 40–49 years | 82.0 (77.7–86.4) | 83.9 (80.9–86.8) | 93.0 (91.5–94.6) | 77.9 (71.6–84.2) | 85.7 (81.0–90.3) | 93.1 (89.6–96.6) |
|  | 50–59 years | 66.5 (59.3–73.6) | 84.1 (80.5–87.7) | 92.6 (90.5–94.7) | 82.1 (78.9–85.2) | 82.3 (79.1–85.4) | 92.2 (89.9–94.6) |
|  | 60–69 years | 56.4 (45.0–67.7) | 69.4 (61.6–77.3) | 89.1 (84.8–93.5) | 65.7 (60.2–71.1) | 70.8 (65.6–76.1) | 89.5 (85.9–93.1) |
|  | 70–80 years | 19.2 (-5.0–43.5) | 66.3 (41.4–91.2) | 89.3 (75.9–102.6) | 47.3 (39.3–55.2) | 63.5 (52.3–74.6) | 91.4 (84.0–98.7) |
|  | Days primary activity missed (mean, 95% CI) | 2.46 (2.09–2.82) | 1.87 (1.66–2.09) | 1.47 (1.33–1.61) | 3.97 (3.62–4.31) | 2.81 (2.53–3.10) | 2.64 (2.33–2.95) |
|  | Days lying in bed (mean, 95% CI) | 0.48 (0.35–0.61) | 0.24 (0.16–0.32) | 0.21 (0.16–0.25) | 0.87 (0.72–1.02) | 0.42 (0.34–0.50) | 0.41 (0.32–0.50) |
| Notes | |  |  |  |  |  |  |
| CI: Confidence interval; OOPE: Out-of-pocket expenditure | | | |  |  |  |  |
| 10-year CVD risk defines as the chance of developing a cardiovascular event over 10 years | | | | |  |  |  |
| ^a)^ in the last four weeks | |  |  |  |  |  |  |
| ^b)^ in the last 12 months | |  |  |  |  |  |  |
| ^c)^ OOPE were only asked to those who utilised outpatient and/or inpatient services. | | | | |  |  |  |
| ^d)^ The percentages were calculated based on the total number of respondents by aged groups. | | | | |  |  |  |
| OOPE medical expenses were converted to 2014 US Dollars (US$). | | | |  |  |  |  |
| Bootstrapping with 400 times replications was performed to estimate the standard error. | | | | |  |  |  |

**Table A4. Association between physical activity and CVDs and risk factors for CVDs**

| **Variables** | | **NCD risk** | | | | | | | |
| --- | --- | --- | --- | --- | --- | --- | --- | --- | --- |
|  |  | **Obesity** | | **Hypertension** | | **Diabetes** | | **CVDs** | |
|  |  | AOR | (95% CI) | AOR | (95% CI) | AOR | (95% CI) | AOR | (95% CI) |
| Physical activity (ref. high) | | |  |  |  |  |  |  |  |
|  | Medium | 1.33*** | (1.08–1.65) | 1.25*** | (1.06–1.46) | 1.67** | (1.03–2.70) | 1.27 | (0.88–1.82) |
|  | Low | 1.48*** | (1.18–1.87) | 1.20** | (1.01–1.43) | 1.70** | (1.03–2.78) | 1.44** | (1.00–2.10) |
| Period (ref. 2007) | |  |  |  |  |  |  |  |  |
|  | 2014 | 3.29*** | (2.69–4.02) | 1.61*** | (1.40–1.85) | 7.01*** | (4.65–10.57) | 2.35*** | (1.70–3.25) |
| Sex (ref. male) | |  |  |  |  |  |  |  |  |
|  | Female | 7.56*** | (5.06–11.29) | 1.35** | (1.05–1.74) | 0.49** | (0.26–0.92) | 0.72 | (0.45–1.14) |
| Age (ref. 40–49 years) | |  |  |  |  |  |  |  |  |
|  | 50–59 years | 0.51*** | (0.41–0.64) | 1.82*** | (1.54–2.16) | 1.82** | (1.08–3.07) | 1.08 | (0.73–1.59) |
|  | 60–69 years | 0.17*** | (0.12–0.23) | 3.86*** | (3.06–4.88) | 2.26** | (1.18–4.35) | 1.40 | (0.89–2.22) |
|  | 70–80 years | 0.03*** | (0.02–0.05) | 7.76*** | (5.50–10.95) | 1.00 | (0.40–2.47) | 1.42 | (0.78–2.59) |
| Marital status (ref. not currently married) | | | |  |  |  |  |  |  |
|  | Currently married | 2.16*** | (1.63–2.87) | 0.81** | (0.66–1.00) | 1.44 | (0.83–2.50) | 0.76 | (0.52–1.11) |
| Ethnicity (ref. Javanese) | | |  |  |  |  |  |  |  |
|  | Sundanese | 0.59*** | (0.41–0.86) | 1.43** | (1.09–1.89) | 0.50* | (0.24–1.04) | 1.23 | (0.76–1.99) |
|  | Others | 0.64*** | (0.48–0.84) | 0.82* | (0.67–1.02) | 0.84 | (0.48–1.46) | 1.20 | (0.81–1.77) |
| Education (ref. no education) | | |  |  |  |  |  |  |  |
|  | No education | 2.08*** | (1.58–2.73) | 0.94 | (0.77–1.14) | 1.63* | (0.94–2.82) | 1.48** | (1.01–2.17) |
|  | Primary | 2.58*** | (1.71–3.88) | 0.71** | (0.53–0.96) | 2.79*** | (1.37–5.68) | 2.07*** | (1.24–3.48) |
|  | Junior high school | 5.52*** | (3.57–8.55) | 0.85 | (0.63–1.13) | 1.60 | (0.77–3.29) | 1.57* | (0.93–2.64) |
|  | Senior high school | 17.01*** | (8.82–32.82) | 1.06 | (0.69–1.63) | 2.85** | (1.12–7.23) | 1.87* | (0.91–3.87) |
| Type of work (ref. unemployed) | | |  |  |  |  |  |  |  |
|  | Casual worker | 0.91 | (0.68–1.23) | 0.61*** | (0.49–0.76) | 0.22*** | (0.11–0.43) | 0.38*** | (0.24–0.61) |
|  | Self-employed | 1.20 | (0.92–1.56) | 0.65*** | (0.53–0.80) | 0.40*** | (0.25–0.66) | 0.40*** | (0.27–0.58) |
|  | Government/private worker | 2.03*** | (1.44–2.85) | 0.98 | (0.76–1.26) | 0.36*** | (0.19–0.69) | 0.31*** | (0.18–0.52) |
| Residency (ref. rural) | |  |  |  |  |  |  |  |  |
|  | Urban | 4.99*** | (3.83–6.50) | 1.22** | (1.03–1.45) | 3.24*** | (1.97–5.33) | 1.22 | (0.87–1.70) |
| Region of residency (ref. Java–Bali) | | | |  |  |  |  |  |  |
|  | Sumatera | 2.71*** | (1.94–3.77) | 1.25* | (0.99–1.59) | 0.52* | (0.26–1.02) | 1.21 | (0.79–1.85) |
|  | Nusa Tenggara | 0.47*** | (0.28–0.81) | 0.96 | (0.66–1.42) | 0.37 | (0.12–1.21) | 0.51* | (0.23–1.12) |
|  | Kalimantan | 1.15 | (0.64–2.07) | 2.72*** | (1.75–4.23) | 1.37 | (0.46–4.07) | 0.61 | (0.26–1.41) |
|  | Sulawesi | 0.39*** | (0.21–0.73) | 1.64** | (1.05–2.58) | 0.47 | (0.13–1.66) | 0.56 | (0.25–1.25) |
| PCE (ref. Q1) | |  |  |  |  |  |  |  |  |
|  | Q2 | 1.18 | (0.90–1.55) | 0.95 | (0.77–1.16) | 3.24*** | (1.61–6.52) | 1.13 | (0.69–1.85) |
|  | Q3 | 1.93*** | (1.45–2.57) | 1.03 | (0.83–1.28) | 2.45** | (1.20–5.02) | 2.01*** | (1.25–3.23) |
|  | Q4 | 2.49*** | (1.84–3.36) | 1.14 | (0.91–1.42) | 3.94*** | (1.92–8.06) | 2.11*** | (1.30–3.42) |
|  | Q5 | 3.40*** | (2.44–4.74) | 1.37** | (1.07–1.76) | 7.27*** | (3.52–15.01) | 2.06*** | (1.24–3.45) |
| Insurance coverage (ref. uninsured) | | |  |  |  |  |  |  |  |
|  | Insured | 1.05 | (0.87–1.29) | 1.04 | (0.90–1.21) | 1.30 | (0.86–1.96) | 1.39** | (1.02–1.88) |
| Tobacco use (ref. non-smoker) | | |  |  |  |  |  |  |  |
|  | Former smoker | 0.49*** | (0.32–0.75) | 0.94 | (0.67–1.30) | 1.18 | (0.57–2.42) | 2.26*** | (1.33–3.85) |
|  | Light smoker | 0.23*** | (0.15–0.35) | 0.67*** | (0.50–0.91) | 0.25*** | (0.11–0.58) | 0.55* | (0.30–1.01) |
|  | Moderate | 0.15*** | (0.10–0.22) | 0.57*** | (0.43–0.74) | 0.15*** | (0.07–0.35) | 0.26*** | (0.14–0.49) |
|  | Heavy | 0.12*** | (0.07–0.21) | 0.55*** | (0.38–0.81) | 0.11*** | (0.03–0.44) | 0.14*** | (0.05–0.45) |
| Food consumption last week | | |  |  |  |  |  |  |  |
|  | Fruit | 1.01 | (0.98–1.04) | 1.00 | (0.98–1.02) | 1.02 | (0.97–1.07) | 1.03 | (0.99–1.07) |
|  | Vegetables | 1.02 | (0.99–1.05) | 1.03** | (1.00–1.05) | 1.01 | (0.95–1.07) | 0.97 | (0.93–1.01) |
|  | Meat | 1.10*** | (1.04–1.16) | 0.94*** | (0.91–0.98) | 1.00 | (0.90–1.11) | 0.96 | (0.88–1.05) |
|  | Fish | 1.01 | (0.96–1.05) | 0.97 | (0.94–1.01) | 0.95 | (0.88–1.03) | 1.00 | (0.94–1.06) |
|  | Dairy | 1.01 | (0.97–1.05) | 0.97** | (0.94–1.00) | 1.01 | (0.93–1.09) | 1.06* | (1.00–1.12) |
| Notes: | |  |  |  |  |  |  |  |  |
| AOR: Adjusted Odds Ratio; CI: Confidence Interval; PCE: Per capita expenditure | | | | | | |  |  |  |
| AOR was estimated using multilevel logistic regression | | | | |  |  |  |  |  |
| *** p<0.01, ** p<0.05, * p<0.1 | | |  |  |  |  |  |  |  |

**Table A5. Association between physical activity and 10-year CVD risk**

| **Variables** | | **10 Year CVD risk** | | | | | |
| --- | --- | --- | --- | --- | --- | --- | --- |
|  |  | **Low (5% to <10%)** | | **Moderate (10% to <20%)** | | **High (≥20%)** | |
|  |  | AOR | (95% CI) | AOR | (95% CI) | AOR | (95% CI) |
| Physical activity (ref. high) | |  |  |  |  |  |  |
|  | Medium | 0.97 | (0.87–1.07) | 1.10 | (0.95–1.27) | 1.18 | (0.84–1.65) |
|  | Low | 0.84*** | (0.75–0.94) | 1.23*** | (1.05–1.43) | 2.11*** | (1.51–2.95) |
| Period (ref. 2007) | |  |  |  |  |  |  |
|  | 2014 | 1.33*** | (1.22–1.46) | 2.65*** | (2.33–3.02) | 4.70*** | (3.44–6.42) |
| Marital status (ref. not currently married) | | |  |  |  |  |  |
|  | Currently married | 1.39*** | (1.24–1.56) | 0.55*** | (0.47–0.64) | 0.23*** | (0.17–0.31) |
| Ethnicity (ref. Javanese) | |  |  |  |  |  |  |
|  | Sundanese | 1.10 | (0.95–1.27) | 1.12 | (0.91–1.39) | 0.76 | (0.47–1.23) |
|  | Others | 1.15** | (1.03–1.30) | 0.83** | (0.70–0.98) | 0.74 | (0.51–1.08) |
| Education (ref. no education) | | |  |  |  |  |  |
|  | No education | 1.00 | (0.89–1.11) | 0.87* | (0.75–1.02) | 0.50*** | (0.36–0.71) |
|  | Primary | 0.93 | (0.79–1.10) | 0.61*** | (0.48–0.79) | 0.27*** | (0.15–0.49) |
|  | Junior high school | 0.84** | (0.72–0.98) | 0.68*** | (0.54–0.86) | 0.23*** | (0.12–0.42) |
|  | Senior high school | 0.83 | (0.65–1.05) | 0.66** | (0.46–0.95) | 0.28** | (0.10–0.77) |
| Type of work (ref. unemployed) | | |  |  |  |  |  |
|  | Casual worker | 1.43*** | (1.24–1.64) | 0.44*** | (0.36–0.53) | 0.29*** | (0.19–0.43) |
|  | Self-employed | 1.60*** | (1.42–1.81) | 0.75*** | (0.64–0.87) | 0.51*** | (0.37–0.69) |
|  | Government/private worker | 1.74*** | (1.50–2.03) | 0.40*** | (0.32–0.49) | 0.06*** | (0.03–0.13) |
| Residency (ref. rural) | |  |  |  |  |  |  |
|  | Urban | 1.09* | (0.99–1.20) | 1.01 | (0.87–1.16) | 0.81 | (0.60–1.09) |
| Region of residency (ref. Java–Bali) | | |  |  |  |  |  |
|  | Sumatera | 0.99 | (0.88–1.12) | 1.15 | (0.95–1.39) | 1.02 | (0.68–1.53) |
|  | Nusa Tenggara | 0.70*** | (0.57–0.86) | 1.38** | (1.02–1.87) | 0.85 | (0.44–1.65) |
|  | Kalimantan | 0.87 | (0.69–1.09) | 1.23 | (0.87–1.73) | 1.16 | (0.56–2.41) |
|  | Sulawesi | 0.94 | (0.74–1.19) | 0.93 | (0.65–1.33) | 0.94 | (0.45–1.98) |
| PCE (ref. Q1) | |  |  |  |  |  |  |
|  | Q2 | 1.06 | (0.93–1.21) | 0.93 | (0.78–1.11) | 0.72* | (0.50–1.05) |
|  | Q3 | 1.04 | (0.90–1.19) | 0.93 | (0.78–1.13) | 0.88 | (0.60–1.29) |
|  | Q4 | 1.12 | (0.97–1.29) | 0.93 | (0.76–1.13) | 0.85 | (0.56–1.27) |
|  | Q5 | 1.21** | (1.04–1.41) | 0.85 | (0.69–1.05) | 0.88 | (0.56–1.39) |
| Insurance coverage (ref. uninsured) | | |  |  |  |  |  |
|  | Insured | 1.05 | (0.96–1.15) | 0.93 | (0.82–1.06) | 1.12 | (0.85–1.47) |
| Food consumption last week | | |  |  |  |  |  |
|  | Fruit | 1.00 | (0.99–1.01) | 1.01 | (0.99–1.02) | 1.01 | (0.97–1.05) |
|  | Vegetables | 1.01 | (0.99–1.02) | 1.00 | (0.99–1.02) | 0.98 | (0.95–1.02) |
|  | Meat | 1.00 | (0.97–1.02) | 0.98 | (0.95–1.02) | 0.89*** | (0.81–0.97) |
|  | Fish | 1.01 | (0.99–1.03) | 0.97** | (0.95–1.00) | 0.96 | (0.91–1.01) |
|  | Dairy | 0.99 | (0.97–1.01) | 1.01 | (0.98–1.04) | 1.08** | (1.01–1.14) |
| Notes: | |  |  |  |  |  |  |
| AOR: Adjusted Odds Ratio; CI: Confidence Interval; PCE: Per capita expenditure | | | | | |  |  |
| AOR was estimated using multilevel logistic regression | | | |  |  |  |  |
| *** p<0.01, ** p<0.05, * p<0.1 | | |  |  |  |  |  |

**Table A6. Association between physical activity and health service use**

| **Variables** | | **Health service use** | | | | | | | | |
| --- | --- | --- | --- | --- | --- | --- | --- | --- | --- | --- |
|  |  | **Outpatient** | | | |  | **Inpatient** | | | |
|  |  | **Any visit** | | **Number of visit** | |  | **Any visit** | | **Number of visit** | |
|  |  | AOR | (95% CI) | IRR | (95% CI) |  | AOR | (95% CI) | IRR | (95% CI) |
| Physical activity (ref. high) | | |  |  |  |  |  |  |  |  |
|  | Medium | 1.04 | (0.91–1.18) | 1.01 | (0.90–1.14) |  | 0.93 | (0.68–1.28) | 0.98 | (0.72–1.34) |
|  | Low | 1.26*** | (1.10–1.44) | 1.31*** | (1.15–1.49) |  | 1.45** | (1.07–1.96) | 1.44** | (1.06–1.95) |
| Period (ref. 2007) | |  |  |  |  |  |  |  |  |  |
|  | 2014 | 1.26*** | (1.12–1.42) | 1.32*** | (1.18–1.48) |  | 1.70*** | (1.29–2.25) | 1.81*** | (1.37–2.39) |
| Sex (ref. male) | |  |  |  |  |  |  |  |  |  |
|  | Female | 1.30*** | (1.10–1.53) | 1.19** | (1.02–1.40) |  | 0.89 | (0.62–1.26) | 0.85 | (0.60–1.21) |
| Age (ref. 40–49 years) | | |  |  |  |  |  |  |  |  |
|  | 50–59 years | 0.97 | (0.85–1.12) | 0.95 | (0.84–1.08) |  | 1.11 | (0.80–1.54) | 1.13 | (0.81–1.56) |
|  | 60–69 years | 1.10 | (0.94–1.30) | 1.03 | (0.89–1.21) |  | 1.15 | (0.79–1.68) | 1.18 | (0.80–1.72) |
|  | 70–80 years | 1.07 | (0.85–1.34) | 1.00 | (0.80–1.23) |  | 1.42 | (0.88–2.28) | 1.39 | (0.87–2.23) |
| Marital status (ref. not currently married) | | | |  |  |  |  |  |  |  |
|  | Currently married | 1.15** | (1.00–1.33) | 1.19** | (1.04–1.36) |  | 1.27 | (0.94–1.72) | 1.19 | (0.88–1.61) |
| Ethnicity (ref. Javanese) | | |  |  |  |  |  |  |  |  |
|  | Sundanese | 0.96 | (0.80–1.14) | 0.97 | (0.82–1.15) |  | 0.92 | (0.63–1.34) | 0.94 | (0.64–1.37) |
|  | Others | 0.92 | (0.80–1.06) | 0.94 | (0.82–1.07) |  | 0.91 | (0.67–1.23) | 0.93 | (0.68–1.26) |
| Education (ref. no education) | | |  |  |  |  |  |  |  |  |
|  | No education | 0.94 | (0.82–1.08) | 0.90* | (0.79–1.02) |  | 0.83 | (0.61–1.13) | 0.82 | (0.60–1.11) |
|  | Primary | 0.94 | (0.77–1.15) | 0.97 | (0.81–1.17) |  | 0.82 | (0.53–1.25) | 0.91 | (0.60–1.38) |
|  | Junior high school | 0.97 | (0.80–1.17) | 0.85* | (0.71–1.02) |  | 0.85 | (0.57–1.27) | 0.85 | (0.57–1.27) |
|  | Senior high school | 0.95 | (0.73–1.25) | 0.74** | (0.57–0.97) |  | 0.65 | (0.38–1.13) | 0.66 | (0.38–1.15) |
| Type of work (ref. unemployed) | | |  |  |  |  |  |  |  |  |
|  | Casual worker | 0.78*** | (0.65–0.92) | 0.75*** | (0.64–0.88) |  | 0.40*** | (0.26–0.62) | 0.38*** | (0.25–0.59) |
|  | Self-employed | 0.86** | (0.74–0.99) | 0.85** | (0.75–0.97) |  | 0.75* | (0.56–1.01) | 0.67*** | (0.50–0.90) |
|  | Government/private worker | 0.79** | (0.65–0.95) | 0.80*** | (0.67–0.95) |  | 0.79 | (0.54–1.17) | 0.78 | (0.54–1.14) |
| Residency (ref. rural) | |  |  |  |  |  |  |  |  |  |
|  | Urban | 0.90* | (0.80–1.02) | 0.93 | (0.83–1.04) |  | 1.03 | (0.79–1.33) | 1.04 | (0.80–1.34) |
| Region of residency (ref. Java–Bali) | | | |  |  |  |  |  |  |  |
|  | Sumatera | 0.88* | (0.75–1.02) | 0.97 | (0.84–1.13) |  | 1.31 | (0.95–1.80) | 1.36* | (0.99–1.88) |
|  | Nusa Tenggara | 0.83 | (0.64–1.08) | 0.78** | (0.61–1.00) |  | 1.25 | (0.73–2.13) | 1.30 | (0.77–2.21) |
|  | Kalimantan | 0.81 | (0.61–1.07) | 0.90 | (0.69–1.17) |  | 1.08 | (0.59–1.97) | 1.00 | (0.54–1.83) |
|  | Sulawesi | 0.57*** | (0.42–0.77) | 0.50*** | (0.37–0.68) |  | 0.91 | (0.49–1.70) | 0.90 | (0.49–1.66) |
| PCE (ref. Q1) | |  |  |  |  |  |  |  |  |  |
|  | Q2 | 1.35*** | (1.14–1.60) | 1.34*** | (1.14–1.58) |  | 1.01 | (0.68–1.50) | 0.94 | (0.64–1.39) |
|  | Q3 | 1.50*** | (1.26–1.78) | 1.50*** | (1.27–1.77) |  | 1.20 | (0.82–1.78) | 1.23 | (0.84–1.80) |
|  | Q4 | 1.59*** | (1.33–1.91) | 1.60*** | (1.35–1.89) |  | 1.20 | (0.80–1.80) | 1.22 | (0.82–1.81) |
|  | Q5 | 1.68*** | (1.38–2.03) | 1.69*** | (1.41–2.02) |  | 2.02*** | (1.36–3.01) | 2.02*** | (1.37–2.97) |
| Insurance coverage (ref. uninsured) | | | |  |  |  |  |  |  |  |
|  | Insured | 1.39*** | (1.24–1.56) | 1.43*** | (1.29–1.59) |  | 2.22*** | (1.73–2.85) | 1.98*** | (1.55–2.54) |
| Tobacco use (ref. non-smoker) | | |  |  |  |  |  |  |  |  |
|  | Former smoker | 1.43*** | (1.14–1.79) | 1.30** | (1.05–1.61) |  | 1.38 | (0.90–2.10) | 1.36 | (0.90–2.05) |
|  | Light smoker | 0.76** | (0.60–0.95) | 0.75** | (0.61–0.93) |  | 0.53** | (0.31–0.91) | 0.50*** | (0.29–0.85) |
|  | Moderate | 0.72*** | (0.59–0.87) | 0.69*** | (0.57–0.83) |  | 0.52*** | (0.33–0.82) | 0.54*** | (0.35–0.84) |
|  | Heavy | 0.67*** | (0.49–0.90) | 0.66*** | (0.50–0.88) |  | 0.48** | (0.24–0.96) | 0.45** | (0.22–0.91) |
| Food consumption last week | | |  |  |  |  |  |  |  |  |
|  | Fruit | 1.00 | (0.98–1.01) | 1.01 | (1.00–1.02) |  | 1.01 | (0.97–1.04) | 1.00 | (0.96–1.03) |
|  | Vegetables | 1.00 | (0.98–1.01) | 1.00 | (0.98–1.01) |  | 0.99 | (0.96–1.03) | 0.98 | (0.95–1.02) |
|  | Meat | 1.02 | (0.99–1.05) | 1.01 | (0.98–1.04) |  | 1.01 | (0.94–1.08) | 1.00 | (0.94–1.08) |
|  | Fish | 1.02** | (1.00–1.05) | 1.02 | (1.00–1.04) |  | 0.99 | (0.94–1.04) | 1.00 | (0.95–1.05) |
|  | Dairy | 1.06*** | (1.03–1.08) | 1.05*** | (1.03–1.08) |  | 1.04 | (0.99–1.09) | 1.04 | (0.99–1.09) |
| BMI | | 1.00 | (0.99–1.01) | 0.99 | (0.98–1.01) |  | 0.97** | (0.94–1.00) | 0.96** | (0.94–0.99) |
| Number of NCDs | |  |  |  |  |  |  |  |  |  |
|  | Single NCD | 1.37*** | (1.21–1.55) | 1.39*** | (1.24–1.57) |  | 1.04 | (0.77–1.39) | 1.04 | (0.78–1.40) |
|  | 2 NCDs | 2.48*** | (2.12–2.91) | 2.54*** | (2.19–2.94) |  | 1.98*** | (1.41–2.79) | 2.26*** | (1.62–3.15) |
|  | 3+ NCDs | 4.70*** | (3.72–5.94) | 4.25*** | (3.45–5.23) |  | 4.69*** | (3.09–7.13) | 4.67*** | (3.10–7.02) |
| Notes: | |  |  |  |  |  |  |  |  |  |
| AOR: Adjusted Odds Ratio; CI: Confidence Interval; PCE: Per capita expenditure | | | | | | | |  |  |  |
| AOR was estimated using multilevel logistic regression | | | | |  |  |  |  |  |  |
| IRR was estimated using multilevel negative binomial regression | | | | | |  |  |  |  |  |
| *** p<0.01, ** p<0.05, * p<0.1 | | |  |  |  |  |  |  |  |  |

**Table A7. Association between physical activity and catastrophic health expenditure**

| **Variables** | | **Catastrophic Health Expenditure** | | | | | |
| --- | --- | --- | --- | --- | --- | --- | --- |
|  |  | **10% of total  household expenditure** | | **25% of total household expenditure** | | **40% of non-food expenditure** | |
|  |  | AOR | (95% CI) | AOR | (95% CI) | AOR | (95% CI) |
| Physical activity (ref. high) | |  |  |  |  |  |  |
|  | Medium | 1.39** | (1.04–1.88) | 1.45 | (0.82–2.54) | 1.44 | (0.90–2.31) |
|  | Low | 1.66*** | (1.21–2.28) | 1.61 | (0.88–2.96) | 1.89** | (1.15–3.12) |
| Period (ref. 2007) | |  |  |  |  |  |  |
|  | 2014 | 1.55*** | (1.19–2.01) | 0.73 | (0.44–1.20) | 0.85 | (0.56–1.29) |
| Sex (ref. male) | |  |  |  |  |  |  |
|  | Female | 1.10 | (0.75–1.61) | 0.94 | (0.47–1.88) | 1.27 | (0.71–2.27) |
| Age (ref. 40–49 years) | |  |  |  |  |  |  |
|  | 50–59 years | 1.24 | (0.90–1.72) | 1.91** | (1.01–3.62) | 1.39 | (0.83–2.36) |
|  | 60–69 years | 1.50* | (0.99–2.26) | 3.64*** | (1.68–7.89) | 2.84*** | (1.50–5.38) |
|  | 70–80 years | 1.20 | (0.68–2.12) | 2.05 | (0.67–6.28) | 2.08 | (0.86–5.06) |
| Marital status (ref. not currently married) | | |  |  |  |  |  |
|  | Currently married | 1.37* | (0.96–1.96) | 1.53 | (0.79–2.95) | 1.95** | (1.11–3.45) |
| Ethnicity (ref. Javanese) | |  |  |  |  |  |  |
|  | Sundanese | 0.76 | (0.47–1.24) | 1.58 | (0.69–3.58) | 0.91 | (0.44–1.89) |
|  | Others | 0.74 | (0.50–1.08) | 1.10 | (0.55–2.21) | 0.72 | (0.40–1.29) |
| Education (ref. no education) | |  |  |  |  |  |  |
|  | No education | 0.94 | (0.68–1.31) | 1.04 | (0.56–1.92) | 0.98 | (0.59–1.63) |
|  | Primary | 0.95 | (0.59–1.54) | 1.50 | (0.65–3.44) | 0.86 | (0.41–1.81) |
|  | Junior high school | 0.85 | (0.53–1.38) | 1.00 | (0.41–2.41) | 0.66 | (0.30–1.42) |
|  | Senior high school | 0.60 | (0.29–1.24) | 0.39 | (0.09–1.75) | 0.31* | (0.09–1.13) |
| Type of work (ref. unemployed) | |  |  |  |  |  |  |
|  | Casual worker | 0.63** | (0.42–0.94) | 0.54 | (0.25–1.16) | 0.49** | (0.26–0.92) |
|  | Self-employed | 0.79 | (0.57–1.09) | 0.61* | (0.34–1.10) | 0.69 | (0.42–1.13) |
|  | Government/private worker | 0.59** | (0.38–0.93) | 0.59 | (0.26–1.35) | 0.63 | (0.31–1.26) |
| Residency (ref. rural) | |  |  |  |  |  |  |
|  | Urban | 0.88 | (0.64–1.21) | 0.82 | (0.45–1.48) | 0.78 | (0.47–1.29) |
| Region of residency (ref. Java–Bali) | | |  |  |  |  |  |
|  | Sumatera | 0.63* | (0.40–1.00) | 0.32** | (0.13–0.79) | 0.71 | (0.36–1.41) |
|  | Nusa Tenggara | 0.79 | (0.36–1.70) | 0.70 | (0.18–2.67) | 0.58 | (0.17–1.99) |
|  | Kalimantan | 0.74 | (0.33–1.69) | 0.52 | (0.11–2.38) | 0.62 | (0.17–2.27) |
|  | Sulawesi | 1.00 | (0.45–2.26) | 0.67 | (0.15–2.92) | 1.11 | (0.32–3.77) |
| PCE (ref. Q1) | |  |  |  |  |  |  |
|  | Q2 | 1.34 | (0.86–2.10) | 1.51 | (0.62–3.71) | 1.39 | (0.70–2.79) |
|  | Q3 | 2.12*** | (1.35–3.32) | 2.41** | (1.00–5.77) | 1.89* | (0.94–3.79) |
|  | Q4 | 2.45*** | (1.55–3.88) | 3.85*** | (1.56–9.49) | 2.62** | (1.26–5.48) |
|  | Q5 | 4.88*** | (3.02–7.88) | 9.15*** | (3.58–23.43) | 8.74*** | (4.06–18.78) |
| Insurance coverage (ref. uninsured) | | |  |  |  |  |  |
|  | Insured | 1.13 | (0.86–1.49) | 1.09 | (0.65–1.83) | 1.03 | (0.67–1.59) |
| Tobacco use (ref. non-smoker) | |  |  |  |  |  |  |
|  | Former smoker | 1.55* | (0.93–2.56) | 1.45 | (0.57–3.66) | 1.74 | (0.81–3.76) |
|  | Light smoker | 1.32 | (0.80–2.18) | 0.62 | (0.23–1.68) | 0.96 | (0.43–2.13) |
|  | Moderate | 1.10 | (0.70–1.73) | 0.92 | (0.40–2.11) | 1.23 | (0.62–2.43) |
|  | Heavy | 0.67 | (0.33–1.38) | 0.77 | (0.22–2.68) | 0.60 | (0.20–1.84) |
| Food consumption last week | |  |  |  |  |  |  |
|  | Fruit | 0.98 | (0.94–1.01) | 1.00 | (0.94–1.07) | 0.97 | (0.92–1.03) |
|  | Vegetables | 1.02 | (0.98–1.06) | 1.00 | (0.93–1.08) | 1.00 | (0.94–1.06) |
|  | Meat | 1.01 | (0.94–1.09) | 0.88* | (0.76–1.02) | 0.93 | (0.82–1.05) |
|  | Fish | 0.97 | (0.92–1.02) | 0.97 | (0.88–1.07) | 0.97 | (0.90–1.06) |
|  | Dairy | 1.05* | (0.99–1.10) | 1.03 | (0.93–1.14) | 1.05 | (0.96–1.14) |
| BMI | | 0.99 | (0.95–1.02) | 1.01 | (0.95–1.07) | 0.98 | (0.93–1.03) |
| Number of NCDs | |  |  |  |  |  |  |
|  | Single NCD | 0.99 | (0.75–1.32) | 0.71 | (0.42–1.22) | 0.98 | (0.63–1.53) |
|  | 2 NCDs | 1.58** | (1.10–2.26) | 1.35 | (0.70–2.60) | 1.56 | (0.89–2.73) |
|  | 3+ NCDs | 1.86** | (1.09–3.15) | 0.68 | (0.21–2.21) | 0.79 | (0.31–2.05) |
| Notes: | |  |  |  |  |  |  |
| AOR: Adjusted Odds Ratio; CI: Confidence Interval; PCE: Per capita expenditure | | | | |  |  |  |
| AOR was estimated using multilevel logistic regression | | | |  |  |  |  |
| *** p<0.01, ** p<0.05, * p<0.1 | |  |  |  |  |  |  |

**Table A8. Association between physical activity and productivity loss**

| **Variables** | | **Productivity loss** | | | | | |
| --- | --- | --- | --- | --- | --- | --- | --- |
|  |  | **Labour participation** | | **Days primary activity missed** | | **Days stayed in bed** | |
|  |  | AOR | (95% CI) | IRR | (95% CI) | IRR | (95% CI) |
| Physical activity (ref. high) | |  |  |  |  |  |  |
|  | Medium | 0.46*** | (0.38–0.54) | 1.02 | (0.92–1.13) | 1.04 | (0.83–1.29) |
|  | Low | 0.24*** | (0.20–0.28) | 1.23*** | (1.10–1.37) | 1.42*** | (1.12–1.79) |
| Period (ref. 2007) | |  |  |  |  |  |  |
|  | 2014 | 1.29*** | (1.11–1.50) | 1.41*** | (1.28–1.55) | 1.53*** | (1.24–1.88) |
| Sex (ref. male) | |  |  |  |  |  |  |
|  | Female | 0.15*** | (0.12–0.19) | 1.17** | (1.02–1.34) | 1.32* | (1.00–1.75) |
| Age (ref. 40–49 years) | |  |  |  |  |  |  |
|  | 50–59 years | 0.79** | (0.66–0.95) | 1.04 | (0.94–1.16) | 1.18 | (0.94–1.48) |
|  | 60–69 years | 0.26*** | (0.20–0.32) | 1.12* | (0.98–1.27) | 1.22 | (0.92–1.60) |
|  | 70–80 years | 0.09*** | (0.06–0.12) | 1.09 | (0.91–1.31) | 1.72*** | (1.19–2.50) |
| Marital status (ref. not currently married) | | |  |  |  |  |  |
|  | Currently married | 1.01 | (0.84–1.21) | 1.24*** | (1.11–1.39) | 1.09 | (0.86–1.39) |
| Ethnicity (ref. Javanese) | |  |  |  |  |  |  |
|  | Sundanese | 0.46*** | (0.36–0.59) | 1.18** | (1.03–1.36) | 1.22 | (0.92–1.63) |
|  | Others | 0.69*** | (0.57–0.85) | 0.97 | (0.87–1.08) | 1.11 | (0.88–1.39) |
| Education (ref. no education) | |  |  |  |  |  |  |
|  | No education | 0.92 | (0.76–1.11) | 0.93 | (0.83–1.03) | 1.03 | (0.82–1.28) |
|  | Primary | 0.52*** | (0.40–0.69) | 0.95 | (0.82–1.11) | 0.87 | (0.62–1.21) |
|  | Junior high school | 0.79* | (0.60–1.04) | 0.75*** | (0.64–0.87) | 0.54*** | (0.39–0.75) |
|  | Senior high school | 1.63** | (1.07–2.48) | 0.60*** | (0.48–0.76) | 0.37*** | (0.22–0.63) |
| Type of work (ref. unemployed) | |  |  |  |  |  |  |
|  | Casual worker | N/A | N/A | 0.73*** | (0.64–0.84) | 0.63*** | (0.47–0.84) |
|  | Self-employed | N/A | N/A | 0.77*** | (0.69–0.87) | 0.71*** | (0.56–0.91) |
|  | Government/private worker | N/A | N/A | 0.67*** | (0.58–0.78) | 0.63*** | (0.46–0.87) |
| Residency (ref. rural) | |  |  |  |  |  |  |
|  | Urban | 0.39*** | (0.33–0.46) | 0.92* | (0.84–1.00) | 0.87 | (0.72–1.05) |
| Region of residency (ref. Java–Bali) | | |  |  |  |  |  |
|  | Sumatera | 0.99 | (0.79–1.25) | 1.15** | (1.03–1.30) | 1.23 | (0.96–1.58) |
|  | Nusa Tenggara | 1.10 | (0.76–1.58) | 0.99 | (0.82–1.20) | 0.90 | (0.60–1.35) |
|  | Kalimantan | 1.60** | (1.04–2.47) | 0.94 | (0.76–1.17) | 1.15 | (0.74–1.80) |
|  | Sulawesi | 0.51*** | (0.34–0.75) | 1.09 | (0.87–1.36) | 1.28 | (0.82–2.01) |
| PCE (ref. Q1) | |  |  |  |  |  |  |
|  | Q2 | 1.19 | (0.96–1.47) | 1.07 | (0.94–1.22) | 0.81 | (0.62–1.06) |
|  | Q3 | 1.10 | (0.88–1.37) | 1.06 | (0.93–1.22) | 0.92 | (0.70–1.22) |
|  | Q4 | 0.99 | (0.79–1.25) | 1.04 | (0.90–1.19) | 0.91 | (0.68–1.22) |
|  | Q5 | 1.18 | (0.92–1.52) | 1.19** | (1.02–1.38) | 0.88 | (0.65–1.21) |
| Insurance coverage (ref. uninsured) | | |  |  |  |  |  |
|  | Insured | 0.88 | (0.76–1.03) | 1.13*** | (1.04–1.24) | 1.02 | (0.84–1.23) |
| Tobacco use (ref. non-smoker) | |  |  |  |  |  |  |
|  | Former smoker | 0.57*** | (0.41–0.79) | 1.24** | (1.02–1.51) | 1.52** | (1.01–2.28) |
|  | Light smoker | 1.12 | (0.82–1.54) | 1.04 | (0.87–1.24) | 1.23 | (0.85–1.76) |
|  | Moderate | 1.77*** | (1.32–2.38) | 0.87* | (0.75–1.01) | 1.01 | (0.74–1.39) |
|  | Heavy | 1.68** | (1.06–2.68) | 0.91 | (0.73–1.14) | 0.99 | (0.61–1.61) |
| Food consumption last week | |  |  |  |  |  |  |
|  | Fruit | 1.02** | (1.00–1.04) | 1.00 | (0.98–1.01) | 1.03** | (1.00–1.06) |
|  | Vegetables | 0.99 | (0.97–1.02) | 0.99* | (0.97–1.00) | 0.97** | (0.94–0.99) |
|  | Meat | 1.07*** | (1.02–1.12) | 0.99 | (0.96–1.01) | 0.95** | (0.90–1.00) |
|  | Fish | 1.05*** | (1.02–1.09) | 1.01 | (0.99–1.03) | 1.02 | (0.99–1.06) |
|  | Dairy | 0.98 | (0.95–1.01) | 1.03*** | (1.01–1.05) | 1.05** | (1.01–1.10) |
| BMI | | 1.01 | (0.99–1.03) | 0.99** | (0.98–1.00) | 0.96*** | (0.94–0.98) |
| Number of NCDs | |  |  |  |  |  |  |
|  | Single NCD | 0.75*** | (0.64–0.88) | 1.32*** | (1.20–1.46) | 1.26** | (1.03–1.54) |
|  | 2 NCDs | 0.52*** | (0.42–0.64) | 1.94*** | (1.69–2.22) | 2.22*** | (1.68–2.92) |
|  | 3+ NCDs | 0.27*** | (0.20–0.38) | 2.66*** | (2.14–3.31) | 3.38*** | (2.18–5.23) |
| Notes: | |  |  |  |  |  |  |
| AOR: Adjusted Odds Ratio; CI: Confidence Interval; PCE: Per capita expenditure | | | | | |  |  |
| AOR was estimated using multilevel logistic regression | | | |  |  |  |  |
| IRR was estimated using multilevel negative binomial regression | | | | |  |  |  |
| *** p<0.01, ** p<0.05, * p<0.1 | |  |  |  |  |  |  |

**Table A9. Intraclass correlation coefficients (ICC) on outcomes variables**

|  | Overweight/ obesity | Hypertension | Diabetes | CVDs | 10-year risk of CVD (moderate) | 10-year risk of CVD (high) |
| --- | --- | --- | --- | --- | --- | --- |
| Individual |  |  |  |  |  |  |
| Variance (SE) | 27.62 (3.10) | 6.55 (0.49) | 14.76 (2.72) | 4.93 (1.07) | 1.56 (0.22) | 4.53 (1.04) |
| ICC (SE) | 0.89 (0.01) | 0.67 (0.01) | 0.83 (0.01) | 0.64 (0.04) | 0.42 (0.02) | 0.67 (0.03) |
| Household |  |  |  |  |  |  |
| Variance (SE) | 0.0 (0.0) | 0.14 (0.28) | 1.63 (1.28) | 0.82 (0.65) | 0.80 (0.16) | 2.28 (0.76) |
| ICC (SE) | 0.0 (0.0) | 0.01 (0.02) | 0.08 (0.06) | 0.09 (0.07) | 0.14 (0.03) | 0.22 (0.07) |
|  |  |  |  |  |  |  |
|  | Any outpatient visit | Any inpatient visit | CHE:  10% of total household expenditure | CHE:  25% of total household expenditure | CHE:  40% of non-food expenditure | Labour participation |
| Individual |  |  |  |  |  |  |
| Variance (SE) | 0.03 (0.13) | 0.76 (0.57) | 0 (0) | 0 (0) | 0 (0) | 1.73 (0.30) |
| ICC (SE) | 0.12 (0.26) | 0.25 (0.07) | 0.67 (0.03) | 0.71 (0.03) | 0.70 (0.03) | 0.47 (0.02) |
| Household |  |  |  |  |  |  |
| Variance (SE) | 0.45 (0.10) | 0.37 (0.41) | 6.47 (0.87) | 8.10 (1.37) | 7.63 (1.14) | 1.23 (0.25) |
| ICC (SE) | 0.12 (0.03) | 0.08 (0.09) | 0.67 (0.03) | 0.71 (0.03) | 0.70 (0.03) | 0.20 (0.04) |

**Table A10. Robustness check: association between physical activity and CVDs and risk factors for CVDs**

| **Variables** | | **NCD risk** | | | | | | | | |
| --- | --- | --- | --- | --- | --- | --- | --- | --- | --- | --- |
|  |  | **Obesity** | | **Hypertension** | | **Diabetes** | | **CVDs** | | |
|  |  | AOR | (95% CI) | AOR | (95% CI) | AOR | (95% CI) | AOR | (95% CI) |  |
| Physical activity (ref. high) | | |  |  |  |  |  |  |  |  |
|  | Medium | 1.33*** | (1.08–1.65) | 1.25*** | (1.06–1.46) | 1.67** | (1.03–2.70) | 1.27 | (0.88–1.82) |  |
|  | Low | 1.48*** | (1.18–1.87) | 1.20** | (1.01–1.43) | 1.70** | (1.03–2.78) | 1.44** | (1.00–2.10) |  |
| Period (ref. 2007) | |  |  |  |  |  |  |  |  |  |
|  | 2014 | 3.29*** | (2.69–4.02) | 1.61*** | (1.40–1.85) | 7.01*** | (4.65–10.57) | 2.35*** | (1.70–3.25) |  |
| Sex (ref. male) | |  |  |  |  |  |  |  |  |  |
|  | Female | 7.56*** | (5.06–11.29) | 1.35** | (1.05–1.74) | 0.49** | (0.26–0.92) | 0.72 | (0.45–1.14) |  |
| Age (ref. 40–49 years) | | |  |  |  |  |  |  |  |  |
|  | 50–59 years | 0.51*** | (0.41–0.64) | 1.82*** | (1.54–2.16) | 1.82** | (1.08–3.07) | 1.08 | (0.73–1.59) |  |
|  | 60–69 years | 0.17*** | (0.12–0.23) | 3.86*** | (3.06–4.88) | 2.26** | (1.18–4.35) | 1.40 | (0.89–2.22) |  |
|  | 70–80 years | 0.03*** | (0.02–0.05) | 7.76*** | (5.50–10.95) | 1.00 | (0.40–2.47) | 1.42 | (0.78–2.59) |  |
| Marital status (ref. not currently married) | | | |  |  |  |  |  |  |  |
|  | Currently married | 2.16*** | (1.63–2.87) | 0.81** | (0.66–1.00) | 1.44 | (0.83–2.50) | 0.76 | (0.52–1.11) |  |
| Ethnicity (ref. Javanese) | | |  |  |  |  |  |  |  |  |
|  | Sundanese | 0.59*** | (0.41–0.86) | 1.43** | (1.09–1.89) | 0.50* | (0.24–1.04) | 1.23 | (0.76–1.99) |  |
|  | Others | 0.64*** | (0.48–0.84) | 0.82* | (0.67–1.02) | 0.84 | (0.48–1.46) | 1.20 | (0.81–1.77) |  |
| Education (ref. no education) | | |  |  |  |  |  |  |  |  |
|  | No education | 2.08*** | (1.58–2.73) | 0.94 | (0.77–1.14) | 1.63* | (0.94–2.82) | 1.48** | (1.01–2.17) |  |
|  | Primary | 2.58*** | (1.71–3.88) | 0.71** | (0.53–0.96) | 2.79*** | (1.37–5.68) | 2.07*** | (1.24–3.48) |  |
|  | Junior high school | 5.52*** | (3.57–8.55) | 0.85 | (0.63–1.13) | 1.60 | (0.77–3.29) | 1.57* | (0.93–2.64) |  |
|  | Senior high school | 17.01*** | (8.82–32.82) | 1.06 | (0.69–1.63) | 2.85** | (1.12–7.23) | 1.87* | (0.91–3.87) |  |
| Type of work (ref. unemployed) | | |  |  |  |  |  |  |  |  |
|  | Casual worker | 0.91 | (0.68–1.23) | 0.61*** | (0.49–0.76) | 0.22*** | (0.11–0.43) | 0.38*** | (0.24–0.61) |  |
|  | Self-employed | 1.20 | (0.92–1.56) | 0.65*** | (0.53–0.80) | 0.40*** | (0.25–0.66) | 0.40*** | (0.27–0.58) |  |
|  | Government/private worker | 2.03*** | (1.44–2.85) | 0.98 | (0.76–1.26) | 0.36*** | (0.19–0.69) | 0.31*** | (0.18–0.52) |  |
| Residency (ref. rural) | | |  |  |  |  |  |  |  |  |
|  | Urban | 4.99*** | (3.83–6.50) | 1.22** | (1.03–1.45) | 3.24*** | (1.97–5.33) | 1.22 | (0.87–1.70) |  |
| Region of residency (ref. Java–Bali) | | | |  |  |  |  |  |  |  |
|  | Sumatera | 2.71*** | (1.94–3.77) | 1.25* | (0.99–1.59) | 0.52* | (0.26–1.02) | 1.21 | (0.79–1.85) |  |
|  | Nusa Tenggara | 0.47*** | (0.28–0.81) | 0.96 | (0.66–1.42) | 0.37 | (0.12–1.21) | 0.51* | (0.23–1.12) |  |
|  | Kalimantan | 1.15 | (0.64–2.07) | 2.72*** | (1.75–4.23) | 1.37 | (0.46–4.07) | 0.61 | (0.26–1.41) |  |
|  | Sulawesi | 0.39*** | (0.21–0.73) | 1.64** | (1.05–2.58) | 0.47 | (0.13–1.66) | 0.56 | (0.25–1.25) |  |
| PCE (ref. Q1) | |  |  |  |  |  |  |  |  |  |
|  | Q2 | 1.18 | (0.90–1.55) | 0.95 | (0.77–1.16) | 3.24*** | (1.61–6.52) | 1.13 | (0.69–1.85) |  |
|  | Q3 | 1.93*** | (1.45–2.57) | 1.03 | (0.83–1.28) | 2.45** | (1.20–5.02) | 2.01*** | (1.25–3.23) |  |
|  | Q4 | 2.49*** | (1.84–3.36) | 1.14 | (0.91–1.42) | 3.94*** | (1.92–8.06) | 2.11*** | (1.30–3.42) |  |
|  | Q5 | 3.40*** | (2.44–4.74) | 1.37** | (1.07–1.76) | 7.27*** | (3.52–15.01) | 2.06*** | (1.24–3.45) |  |
| Insurance coverage (ref. uninsured) | | | |  |  |  |  |  |  |  |
|  | Insured | 1.05 | (0.87–1.29) | 1.04 | (0.90–1.21) | 1.30 | (0.86–1.96) | 1.39** | (1.02–1.88) |  |
| Tobacco use (ref. non-smoker) | | |  |  |  |  |  |  |  |  |
|  | Former smoker | 0.49*** | (0.32–0.75) | 0.94 | (0.67–1.30) | 1.18 | (0.57–2.42) | 2.26*** | (1.33–3.85) |  |
|  | Light smoker | 0.23*** | (0.15–0.35) | 0.67*** | (0.50–0.91) | 0.25*** | (0.11–0.58) | 0.55* | (0.30–1.01) |  |
|  | Moderate | 0.15*** | (0.10–0.22) | 0.57*** | (0.43–0.74) | 0.15*** | (0.07–0.35) | 0.26*** | (0.14–0.49) |  |
|  | Heavy | 0.12*** | (0.07–0.21) | 0.55*** | (0.38–0.81) | 0.11*** | (0.03–0.44) | 0.14*** | (0.05–0.45) |  |
| Food consumption last week | | |  |  |  |  |  |  |  |  |
|  | Fruit | 1.01 | (0.98–1.04) | 1.00 | (0.98–1.02) | 1.02 | (0.97–1.07) | 1.03 | (0.99–1.07) |  |
|  | Vegetables | 1.02 | (0.99–1.05) | 1.03** | (1.00–1.05) | 1.01 | (0.95–1.07) | 0.97 | (0.93–1.01) |  |
|  | Meat | 1.10*** | (1.04–1.16) | 0.94*** | (0.91–0.98) | 1.00 | (0.90–1.11) | 0.96 | (0.88–1.05) |  |
|  | Fish | 1.01 | (0.96–1.05) | 0.97 | (0.94–1.01) | 0.95 | (0.88–1.03) | 1.00 | (0.94–1.06) |  |
|  | Dairy | 1.01 | (0.97–1.05) | 0.97** | (0.94–1.00) | 1.01 | (0.93–1.09) | 1.06* | (1.00–1.12) |  |
| Notes: | |  |  |  |  |  |  |  |  |  |
| AOR: Adjusted Odds Ratio; CI: Confidence Interval; PCE: Per capita expenditure | | | | | | |  |  |  |  |
| AOR was estimated using multilevel logistic regression | | | | |  |  |  |  |  |  |
| *** p<0.01, ** p<0.05, * p<0.1 | | |  |  |  |  |  |  |  |  |

**Table A11. Robustness check: association between physical activity and health service use**

| **Variables** | | **Health service use** | | | | | | | | |
| --- | --- | --- | --- | --- | --- | --- | --- | --- | --- | --- |
|  |  | **Outpatient** | | | |  | **Inpatient** | | | |
|  |  | **Any visit** | | **Number of visit** | |  | **Any visit** | | **Number of visit** | |
|  |  | AOR | (95% CI) | IRR | (95% CI) |  | AOR | (95% CI) | IRR | (95% CI) |
| Physical activity (ref. high) | | |  |  |  |  |  |  |  |  |
|  | Medium | 1.04 | (0.91–1.18) | 1.01 | (0.90–1.14) |  | 0.93 | (0.68–1.28) | 0.98 | (0.72–1.34) |
|  | Low | 1.26*** | (1.10–1.44) | 1.31*** | (1.15–1.49) |  | 1.45** | (1.07–1.96) | 1.44** | (1.06–1.95) |
| Period (ref. 2007) | |  |  |  |  |  |  |  |  |  |
|  | 2014 | 1.26*** | (1.12–1.42) | 1.32*** | (1.18–1.48) |  | 1.70*** | (1.29–2.25) | 1.81*** | (1.37–2.39) |
| Sex (ref. male) | |  |  |  |  |  |  |  |  |  |
|  | Female | 1.30*** | (1.10–1.53) | 1.19** | (1.02–1.40) |  | 0.89 | (0.62–1.26) | 0.85 | (0.60–1.21) |
| Age (ref. 40–49 years) | | |  |  |  |  |  |  |  |  |
|  | 50–59 years | 0.97 | (0.85–1.12) | 0.95 | (0.84–1.08) |  | 1.11 | (0.80–1.54) | 1.13 | (0.81–1.56) |
|  | 60–69 years | 1.10 | (0.94–1.30) | 1.03 | (0.89–1.21) |  | 1.15 | (0.79–1.68) | 1.18 | (0.80–1.72) |
|  | 70–80 years | 1.07 | (0.85–1.34) | 1.00 | (0.80–1.23) |  | 1.42 | (0.88–2.28) | 1.39 | (0.87–2.23) |
| Marital status (ref. not currently married) | | | |  |  |  |  |  |  |  |
|  | Currently married | 1.15** | (1.00–1.33) | 1.19** | (1.04–1.36) |  | 1.27 | (0.94–1.72) | 1.19 | (0.88–1.61) |
| Ethnicity (ref. Javanese) | | |  |  |  |  |  |  |  |  |
|  | Sundanese | 0.96 | (0.80–1.14) | 0.97 | (0.82–1.15) |  | 0.92 | (0.63–1.34) | 0.94 | (0.64–1.37) |
|  | Others | 0.92 | (0.80–1.06) | 0.94 | (0.82–1.07) |  | 0.91 | (0.67–1.23) | 0.93 | (0.68–1.26) |
| Education (ref. no education) | | |  |  |  |  |  |  |  |  |
|  | No education | 0.94 | (0.82–1.08) | 0.90* | (0.79–1.02) |  | 0.83 | (0.61–1.13) | 0.82 | (0.60–1.11) |
|  | Primary | 0.94 | (0.77–1.15) | 0.97 | (0.81–1.17) |  | 0.82 | (0.53–1.25) | 0.91 | (0.60–1.38) |
|  | Junior high school | 0.97 | (0.80–1.17) | 0.85* | (0.71–1.02) |  | 0.85 | (0.57–1.27) | 0.85 | (0.57–1.27) |
|  | Senior high school | 0.95 | (0.73–1.25) | 0.74** | (0.57–0.97) |  | 0.65 | (0.38–1.13) | 0.66 | (0.38–1.15) |
| Type of work (ref. unemployed) | | |  |  |  |  |  |  |  |  |
|  | Casual worker | 0.78*** | (0.65–0.92) | 0.75*** | (0.64–0.88) |  | 0.40*** | (0.26–0.62) | 0.38*** | (0.25–0.59) |
|  | Self-employed | 0.86** | (0.74–0.99) | 0.85** | (0.75–0.97) |  | 0.75* | (0.56–1.01) | 0.67*** | (0.50–0.90) |
|  | Government/private worker | 0.79** | (0.65–0.95) | 0.80*** | (0.67–0.95) |  | 0.79 | (0.54–1.17) | 0.78 | (0.54–1.14) |
| Residency (ref. rural) | |  |  |  |  |  |  |  |  |  |
|  | Urban | 0.90* | (0.80–1.02) | 0.93 | (0.83–1.04) |  | 1.03 | (0.79–1.33) | 1.04 | (0.80–1.34) |
| Region of residency (ref. Java–Bali) | | | |  |  |  |  |  |  |  |
|  | Sumatera | 0.88* | (0.75–1.02) | 0.97 | (0.84–1.13) |  | 1.31 | (0.95–1.80) | 1.36* | (0.99–1.88) |
|  | Nusa Tenggara | 0.83 | (0.64–1.08) | 0.78** | (0.61–1.00) |  | 1.25 | (0.73–2.13) | 1.30 | (0.77–2.21) |
|  | Kalimantan | 0.81 | (0.61–1.07) | 0.90 | (0.69–1.17) |  | 1.08 | (0.59–1.97) | 1.00 | (0.54–1.83) |
|  | Sulawesi | 0.57*** | (0.42–0.77) | 0.50*** | (0.37–0.68) |  | 0.91 | (0.49–1.70) | 0.90 | (0.49–1.66) |
| PCE (ref. Q1) | |  |  |  |  |  |  |  |  |  |
|  | Q2 | 1.35*** | (1.14–1.60) | 1.34*** | (1.14–1.58) |  | 1.01 | (0.68–1.50) | 0.94 | (0.64–1.39) |
|  | Q3 | 1.50*** | (1.26–1.78) | 1.50*** | (1.27–1.77) |  | 1.20 | (0.82–1.78) | 1.23 | (0.84–1.80) |
|  | Q4 | 1.59*** | (1.33–1.91) | 1.60*** | (1.35–1.89) |  | 1.20 | (0.80–1.80) | 1.22 | (0.82–1.81) |
|  | Q5 | 1.68*** | (1.38–2.03) | 1.69*** | (1.41–2.02) |  | 2.02*** | (1.36–3.01) | 2.02*** | (1.37–2.97) |
| Insurance coverage (ref. uninsured) | | | |  |  |  |  |  |  |  |
|  | Insured | 1.39*** | (1.24–1.56) | 1.43*** | (1.29–1.59) |  | 2.22*** | (1.73–2.85) | 1.98*** | (1.55–2.54) |
| Tobacco use (ref. non-smoker) | | |  |  |  |  |  |  |  |  |
|  | Former smoker | 1.43*** | (1.14–1.79) | 1.30** | (1.05–1.61) |  | 1.38 | (0.90–2.10) | 1.36 | (0.90–2.05) |
|  | Light smoker | 0.76** | (0.60–0.95) | 0.75** | (0.61–0.93) |  | 0.53** | (0.31–0.91) | 0.50*** | (0.29–0.85) |
|  | Moderate | 0.72*** | (0.59–0.87) | 0.69*** | (0.57–0.83) |  | 0.52*** | (0.33–0.82) | 0.54*** | (0.35–0.84) |
|  | Heavy | 0.67*** | (0.49–0.90) | 0.66*** | (0.50–0.88) |  | 0.48** | (0.24–0.96) | 0.45** | (0.22–0.91) |
| Food consumption last week | | |  |  |  |  |  |  |  |  |
|  | Fruit | 1.00 | (0.98–1.01) | 1.01 | (1.00–1.02) |  | 1.01 | (0.97–1.04) | 1.00 | (0.96–1.03) |
|  | Vegetables | 1.00 | (0.98–1.01) | 1.00 | (0.98–1.01) |  | 0.99 | (0.96–1.03) | 0.98 | (0.95–1.02) |
|  | Meat | 1.02 | (0.99–1.05) | 1.01 | (0.98–1.04) |  | 1.01 | (0.94–1.08) | 1.00 | (0.94–1.08) |
|  | Fish | 1.02** | (1.00–1.05) | 1.02 | (1.00–1.04) |  | 0.99 | (0.94–1.04) | 1.00 | (0.95–1.05) |
|  | Dairy | 1.06*** | (1.03–1.08) | 1.05*** | (1.03–1.08) |  | 1.04 | (0.99–1.09) | 1.04 | (0.99–1.09) |
| BMI | | 1.00 | (0.99–1.01) | 0.99 | (0.98–1.01) |  | 0.97** | (0.94–1.00) | 0.96** | (0.94–0.99) |
| Number of NCDs | |  |  |  |  |  |  |  |  |  |
|  | Single NCD | 1.37*** | (1.21–1.55) | 1.39*** | (1.24–1.57) |  | 1.04 | (0.77–1.39) | 1.04 | (0.78–1.40) |
|  | 2 NCDs | 2.48*** | (2.12–2.91) | 2.54*** | (2.19–2.94) |  | 1.98*** | (1.41–2.79) | 2.26*** | (1.62–3.15) |
|  | 3+ NCDs | 4.70*** | (3.72–5.94) | 4.25*** | (3.45–5.23) |  | 4.69*** | (3.09–7.13) | 4.67*** | (3.10–7.02) |
| Notes: | |  |  |  |  |  |  |  |  |  |
| AOR: Adjusted Odds Ratio; CI: Confidence Interval; PCE: Per capita expenditure | | | | | | | |  |  |  |
| AOR was estimated using multilevel logistic regression | | | | |  |  |  |  |  |  |
| IRR was estimated using multilevel negative binomial regression | | | | | |  |  |  |  |  |
| *** p<0.01, ** p<0.05, * p<0.1 | | |  |  |  |  |  |  |  |  |

**Table A12. Robustness check: association between physical activity and catastrophic health expenditure**

| **Variables** | | **Catastrophic Health Expenditure** | | | | | |
| --- | --- | --- | --- | --- | --- | --- | --- |
|  |  | **10% of total  household expenditurea** | | **25% of total household expenditurea** | | **40% of non-food expenditure** | |
|  |  | AOR | (95% CI) | AOR | (95% CI) | AOR | (95% CI) |
| Physical activity (ref. high) | |  |  |  |  |  |  |
|  | Medium | 1.30** | (1.03–1.66) | 1.18 | (0.63–2.22) | 1.15 | (0.77–1.73) |
|  | Low | 1.50*** | (1.15–1.94) | 1.46 | (0.73–2.90) | 1.48* | (0.96–2.26) |
| Period (ref. 2007) | |  |  |  |  |  |  |
|  | 2014 | 1.41*** | (1.12–1.77) | 0.69 | (0.38–1.25) | 0.94 | (0.64–1.38) |
| Sex (ref. male) | |  |  |  |  |  |  |
|  | Female | 0.96 | (0.70–1.31) | 0.91 | (0.40–2.05) | 1.24 | (0.74–2.08) |
| Age (ref. 40–49 years) | |  |  |  |  |  |  |
|  | 50–59 years | 0.93 | (0.72–1.19) | 1.68 | (0.85–3.33) | 1.20 | (0.79–1.84) |
|  | 60–69 years | 1.02 | (0.75–1.40) | 2.70** | (1.13–6.45) | 1.63* | (0.98–2.72) |
|  | 70–80 years | 1.12 | (0.70–1.81) | 2.48 | (0.61–10.04) | 2.09* | (0.99–4.41) |
| Marital status (ref. not currently married) | | |  |  |  |  |  |
|  | Currently married | 0.99 | (0.76–1.31) | 2.08* | (0.90–4.76) | 1.82** | (1.11–2.99) |
| Ethnicity (ref. Javanese) | |  |  |  |  |  |  |
|  | Sundanese | 0.64** | (0.46–0.90) | 0.68 | (0.26–1.74) | 0.61* | (0.35–1.07) |
|  | Others | 0.86 | (0.66–1.12) | 1.06 | (0.50–2.25) | 0.77 | (0.49–1.19) |
| Education (ref. no education) | |  |  |  |  |  |  |
|  | No education | 0.87 | (0.68–1.13) | 1.03 | (0.51–2.07) | 1.11 | (0.74–1.67) |
|  | Primary | 0.84 | (0.58–1.21) | 1.47 | (0.59–3.69) | 1.07 | (0.60–1.90) |
|  | Junior high school | 0.69** | (0.49–0.98) | 0.82 | (0.31–2.14) | 0.68 | (0.38–1.24) |
|  | Senior high school | 0.46*** | (0.28–0.79) | 0.28 | (0.06–1.38) | 0.28** | (0.10–0.80) |
| Type of work (ref. unemployed) | |  |  |  |  |  |  |
|  | Casual worker | 0.70** | (0.50–0.98) | 0.44* | (0.17–1.12) | 0.40*** | (0.22–0.73) |
|  | Self-employed | 0.78* | (0.59–1.03) | 0.56 | (0.27–1.16) | 0.66* | (0.43–1.03) |
|  | Government/private worker | 0.73* | (0.52–1.03) | 0.58 | (0.24–1.44) | 0.75 | (0.43–1.30) |
| Residency (ref. rural) | |  |  |  |  |  |  |
|  | Urban | 0.84 | (0.67–1.05) | 0.88 | (0.46–1.69) | 0.88 | (0.60–1.28) |
| Region of residency (ref. Java–Bali) | | |  |  |  |  |  |
|  | Sumatera | 0.64*** | (0.48–0.87) | 0.20*** | (0.07–0.59) | 0.73 | (0.44–1.19) |
|  | Nusa Tenggara | 0.68 | (0.42–1.11) | 0.69 | (0.18–2.67) | 0.69 | (0.29–1.65) |
|  | Kalimantan | 0.67 | (0.37–1.22) | 0.77 | (0.15–3.97) | 0.93 | (0.36–2.41) |
|  | Sulawesi | 0.70 | (0.35–1.39) | 1.04 | (0.18–5.81) | 1.08 | (0.37–3.15) |
| PCE (ref. Q1) | |  |  |  |  |  |  |
|  | Q2 | 1.28 | (0.91–1.79) | 1.61 | (0.59–4.40) | 1.13 | (0.65–1.96) |
|  | Q3 | 1.63*** | (1.16–2.28) | 2.31 | (0.85–6.28) | 1.33 | (0.76–2.32) |
|  | Q4 | 1.68*** | (1.18–2.40) | 3.70** | (1.33–10.29) | 1.40 | (0.78–2.51) |
|  | Q5 | 2.59*** | (1.81–3.72) | 5.72*** | (2.04–16.10) | 2.95*** | (1.67–5.21) |
| Insurance coverage (ref. uninsured) | | |  |  |  |  |  |
|  | Insured | 1.15 | (0.93–1.42) | 1.49 | (0.83–2.67) | 1.06 | (0.74–1.51) |
| Tobacco use (ref. non-smoker) | |  |  |  |  |  |  |
|  | Former smoker | 1.29 | (0.84–1.98) | 0.97 | (0.29–3.17) | 1.25 | (0.60–2.58) |
|  | Light smoker | 1.12 | (0.76–1.65) | 0.77 | (0.26–2.26) | 1.09 | (0.56–2.11) |
|  | Moderate | 0.82 | (0.58–1.18) | 0.67 | (0.26–1.76) | 1.03 | (0.58–1.84) |
|  | Heavy | 0.87 | (0.52–1.45) | 0.92 | (0.25–3.37) | 0.99 | (0.42–2.31) |
| Food consumption last week | |  |  |  |  |  |  |
|  | Fruit | 0.99 | (0.96–1.02) | 1.02 | (0.95–1.10) | 1.00 | (0.95–1.04) |
|  | Vegetables | 1.02 | (0.99–1.05) | 0.98 | (0.90–1.06) | 0.98 | (0.94–1.04) |
|  | Meat | 1.02 | (0.96–1.08) | 0.91 | (0.77–1.07) | 1.00 | (0.90–1.10) |
|  | Fish | 1.05** | (1.00–1.09) | 1.00 | (0.89–1.12) | 1.00 | (0.93–1.08) |
|  | Dairy | 0.96* | (0.92–1.01) | 0.92 | (0.82–1.04) | 0.95 | (0.89–1.02) |
| BMI | | 0.98 | (0.96–1.01) | 1.03 | (0.96–1.11) | 0.97 | (0.93–1.01) |
| Number of NCDs | |  |  |  |  |  |  |
|  | Single NCD | 0.88 | (0.70–1.11) | 0.54** | (0.29–1.00) | 0.96 | (0.66–1.39) |
|  | 2 NCDs | 1.60*** | (1.20–2.15) | 1.32 | (0.63–2.80) | 1.54* | (0.95–2.51) |
|  | 3+ NCDs | 1.42 | (0.85–2.38) | 0.69 | (0.15–3.18) | 1.24 | (0.52–3.00) |
| Notes: | |  |  |  |  |  |  |
| AOR: Adjusted Odds Ratio; CI: Confidence Interval; PCE: Per capita expenditure | | | | | |  |  |
| AOR was estimated using multilevel logistic regression | | | |  |  |  |  |
| *** p<0.01, ** p<0.05, * p<0.1 | |  |  |  |  |  |  |

**Table A13. Robustness check: association between physical activity and work productivity loss**

| **Variables** | | **Productivity loss** | | | | | |
| --- | --- | --- | --- | --- | --- | --- | --- |
|  |  | **Labour participation** | | **Days primary activity missed** | | **Days stayed in bed** | |
|  |  | AOR | (95% CI) | IRR | (95% CI) | IRR | (95% CI) |
| Physical activity (ref. high) | |  |  |  |  |  |  |
|  | Medium | 0.43*** | (0.34–0.54) | 0.98 | (0.86–1.11) | 1.08 | (0.80–1.47) |
|  | Low | 0.24*** | (0.19–0.31) | 1.10 | (0.95–1.27) | 1.22 | (0.87–1.72) |
| Period (ref. 2007) | |  |  |  |  |  |  |
|  | 2014 | 1.36*** | (1.11–1.65) | 1.42*** | (1.25–1.60) | 1.55 | (0.85–2.81) |
| Sex (ref. male) | |  |  |  |  |  |  |
|  | Female | 0.13*** | (0.09–0.18) | 1.21** | (1.02–1.44) | 1.59** | (1.06–2.38) |
| Age (ref. 40–49 years) | |  |  |  |  |  |  |
|  | 50–59 years | 0.81* | (0.64–1.02) | 1.03 | (0.90–1.17) | 1.13 | (0.83–1.53) |
|  | 60–69 years | 0.23*** | (0.17–0.31) | 1.00 | (0.85–1.19) | 1.10 | (0.70–1.74) |
|  | 70–80 years | 0.08*** | (0.05–0.13) | 0.90 | (0.68–1.18) | 1.41 | (0.78–2.54) |
| Marital status (ref. not currently married) | | |  |  |  |  |  |
|  | Currently married | 1.06 | (0.83–1.36) | 1.24*** | (1.06–1.44) | 1.10 | (0.78–1.55) |
| Ethnicity (ref. Javanese) | |  |  |  |  |  |  |
|  | Sundanese | 0.46*** | (0.33–0.63) | 1.23** | (1.04–1.46) | 1.22 | (0.83–1.79) |
|  | Others | 0.62*** | (0.47–0.81) | 0.92 | (0.80–1.06) | 1.02 | (0.73–1.43) |
| Education (ref. no education) | |  |  |  |  |  |  |
|  | No education | 0.81 | (0.63–1.05) | 0.86** | (0.76–0.99) | 0.92 | (0.68–1.26) |
|  | Primary | 0.42*** | (0.29–0.59) | 0.88 | (0.73–1.08) | 0.68 | (0.28–1.65) |
|  | Junior high school | 0.70** | (0.49–0.99) | 0.81** | (0.67–0.98) | 0.41*** | (0.21–0.81) |
|  | Senior high school | 1.29 | (0.78–2.14) | 0.65*** | (0.49–0.85) | 0.46* | (0.19–1.08) |
| Type of work (ref. unemployed) | |  |  |  |  |  |  |
|  | Casual worker | N/A | N/A | 0.81** | (0.68–0.98) | 0.81 | (0.54–1.22) |
|  | Self-employed | N/A | N/A | 0.88 | (0.75–1.04) | 1.00 | (0.68–1.48) |
|  | Government/private worker | N/A | N/A | 0.78** | (0.64–0.95) | 0.89 | (0.55–1.44) |
| Residency (ref. rural) | |  |  |  |  |  |  |
|  | Urban | 0.34*** | (0.27–0.42) | 0.95 | (0.85–1.07) | 0.93 | (0.72–1.20) |
| Region of residency (ref. Java–Bali) | | |  |  |  |  |  |
|  | Sumatera | 1.22 | (0.90–1.66) | 1.24*** | (1.07–1.45) | 1.42** | (1.02–1.98) |
|  | Nusa Tenggara | 1.34 | (0.85–2.12) | 1.02 | (0.80–1.29) | 0.84 | (0.50–1.42) |
|  | Kalimantan | 2.64*** | (1.34–5.21) | 0.99 | (0.74–1.33) | 1.32 | (0.64–2.72) |
|  | Sulawesi | 0.78 | (0.42–1.45) | 0.99 | (0.71–1.38) | 1.96 | (0.84–4.55) |
| PCE (ref. Q1) | |  |  |  |  |  |  |
|  | Q2 | 1.05 | (0.79–1.40) | 1.19** | (1.01–1.40) | 0.92 | (0.61–1.40) |
|  | Q3 | 0.95 | (0.70–1.27) | 1.10 | (0.93–1.30) | 0.98 | (0.61–1.57) |
|  | Q4 | 0.90 | (0.66–1.22) | 1.07 | (0.89–1.28) | 0.98 | (0.66–1.46) |
|  | Q5 | 1.09 | (0.78–1.52) | 1.16 | (0.96–1.41) | 0.76 | (0.49–1.17) |
| Insurance coverage (ref. uninsured) | | |  |  |  |  |  |
|  | Insured | 0.97 | (0.79–1.18) | 1.16** | (1.03–1.30) | 1.02 | (0.73–1.41) |
| Tobacco use (ref. non-smoker) | |  |  |  |  |  |  |
|  | Former smoker | 0.56** | (0.36–0.88) | 1.19 | (0.92–1.55) | 1.35 | (0.76–2.40) |
|  | Light smoker | 1.08 | (0.71–1.63) | 0.98 | (0.79–1.22) | 1.17 | (0.72–1.91) |
|  | Moderate | 2.01*** | (1.35–2.99) | 0.86 | (0.71–1.04) | 0.97 | (0.59–1.59) |
|  | Heavy | 1.75* | (0.95–3.23) | 1.00 | (0.76–1.31) | 1.36 | (0.61–2.99) |
| Food consumption last week | |  |  |  |  |  |  |
|  | Fruit | 1.02 | (0.99–1.04) | 1.00 | (0.99–1.02) | 1.04** | (1.00–1.08) |
|  | Vegetables | 0.99 | (0.97–1.02) | 1.00 | (0.98–1.01) | 0.98 | (0.93–1.02) |
|  | Meat | 1.06** | (1.00–1.12) | 0.99 | (0.96–1.02) | 0.98 | (0.91–1.06) |
|  | Fish | 0.99 | (0.94–1.03) | 1.02* | (1.00–1.05) | 1.05 | (0.98–1.12) |
|  | Dairy | 1.03 | (0.99–1.07) | 1.02 | (0.99–1.04) | 1.02 | (0.97–1.08) |
| BMI | | 1.01 | (0.99–1.03) | 0.99** | (0.98–1.00) | 0.96*** | (0.94–0.98) |
| Number of NCDs | |  |  |  |  |  |  |
|  | Single NCD | 0.71*** | (0.57–0.87) | 1.35*** | (1.20–1.52) | 1.28* | (0.96–1.70) |
|  | 2 NCDs | 0.58*** | (0.44–0.78) | 1.92*** | (1.61–2.30) | 2.43*** | (1.60–3.69) |
|  | 3+ NCDs | 0.22*** | (0.14–0.37) | 2.36*** | (1.71–3.26) | 2.33** | (1.13–4.79) |
| Notes: | |  |  |  |  |  |  |
| AOR: Adjusted Odds Ratio; CI: Confidence Interval; PCE: Per capita expenditure | | | | | |  |  |
| AOR was estimated using multilevel logistic regression | | | |  |  |  |  |
| IRR was estimated using multilevel negative binomial regression | | | | |  |  |  |
| *** p<0.01, ** p<0.05, * p<0.1 | |  |  |  |  |  |  |
